# A comprehensive survey of space robotic manipulators for on-orbit servicing

**DOI:** 10.3389/frobt.2024.1470950

**Published:** 2024-10-09

**Authors:** Mohammad Alizadeh, Zheng H. Zhu

**Affiliations:** Department of Mechanical Engineering, York University, Toronto, ON, Canada

**Keywords:** on-orbit servicing, space robots, robotic manipulator, motion planning, machine learning, pose estimation, control

## Abstract

On-Orbit Servicing (OOS) robots are transforming space exploration by enabling vital maintenance and repair of spacecraft directly in space. However, achieving precise and safe manipulation in microgravity necessitates overcoming significant challenges. This survey delves into four crucial areas essential for successful OOS manipulation: object state estimation, motion planning, and feedback control. Techniques from traditional vision to advanced X-ray and neural network methods are explored for object state estimation. Strategies for fuel-optimized trajectories, docking maneuvers, and collision avoidance are examined in motion planning. The survey also explores control methods for various scenarios, including cooperative manipulation and handling uncertainties, in feedback control. Additionally, this survey examines how Machine learning techniques can further propel OOS robots towards more complex and delicate tasks in space.

## 1 Introduction

Space exploration has captivated humanity for decades, propelling scientists to answer fundamental questions about the universe. Robots have played a critical role in these endeavors, venturing where humans cannot. However, despite the significant advancements in robotic technology, the field of On-Orbit Servicing (OOS) still faces considerable challenges that must be addressed to enable more complex and delicate tasks in space. These challenges include the precise and safe manipulation of objects in the microgravity environment of space, where uncertainties in object state estimation, motion planning, and feedback control persist. Furthermore, the integration of Machine Learning (ML) techniques in this domain is still in its infancy, presenting a substantial gap in the existing research. This survey aims to fill these gaps by providing a comprehensive overview of current techniques while critically evaluating their effectiveness and identifying the limitations that hinder further progress.

The objectives of this survey are to systematically review the current state-of-the-art techniques in object state estimation, motion planning, and feedback control for OOS robots; to critically evaluate these techniques, highlighting their strengths, weaknesses, and the specific challenges they address; and to identify the areas where further research is needed, particularly in the integration of ML techniques, to enhance the capabilities of OOS robots for future space missions.

While the survey provides a broad overview of existing techniques, it also emphasizes the need for a more nuanced understanding of their limitations. For instance, traditional methods for object state estimation may struggle under certain space conditions, and current motion planning algorithms often have difficulty making real-time adjustments when unexpected obstacles are encountered. Furthermore, the integration of advanced techniques, such as Machine Learning (ML), into the control systems of space robots remains a significant challenge due to the high computational demands and the need for reliable performance in the harsh environment of space. Although various methods have been developed to improve the robustness and accuracy of robotic systems in space, gaps still exist, particularly in the handling of dynamic and unpredictable scenarios. More research is needed to address these gaps and to develop more reliable and efficient systems for future space missions.

**FIGURE 1:**
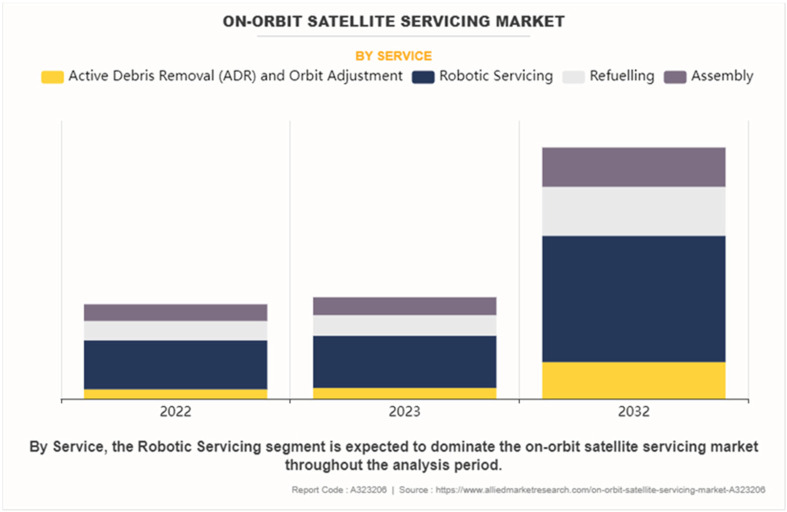
On-orbit satellite servicing market by service. (Mate and Katare, 2024).

According to a recent market analysis (Mate and Katare, 2024), the on-orbit satellite servicing market is expected to experience significant growth, with robotic servicing projected to be the dominant service. This trend underscores the increasing importance of advanced technologies for manipulating and servicing spacecraft in orbit. Figure 1 shows the on-orbit satellite servicing market by service, where the different colors represent various services: Yellow for Active Debris Removal (ADR) and Orbit Adjustment, Dark Blue for Robotic Servicing, Light Grey for Refuelling, and Purple for Assembly. The bars show the projected market size for each service in 2022, 2023, and 2032. As illustrated in Figure 1, the market sizes for various services, including Active Debris Removal (ADR) and Orbit Adjustment, Refuelling, and Assembly, are projected for the years 2022, 2023, and 2032, with Robotic Servicing expected to lead.

The field of OOS robotics is rapidly evolving, demanding a comprehensive understanding of the key technological advancements driving its progress. This survey focuses on critical areas like object state estimation, motion planning, feedback control, and the integration of Machine Learning (ML) techniques. By critically evaluating these advancements, it aims to provide a deeper understanding of their synergistic relationship in propelling OOS robot capabilities. This knowledge will be instrumental in developing future robots capable of performing increasingly complex and delicate tasks in the vast expanse of space.

## 2 A brief history

Space has been the new domain of exploration since the 1950s. Humans have explored space to answer many fundamental questions about the universe. Space robotics has a significant role in human exploration since space is a harsh environment, and using robots to explore space would be much safer and more cost-efficient. This historical exploration has laid the foundation for the technologies that drive current and future on-orbit servicing (OOS) missions, where robotics play an increasingly critical role.

“Two attributes are often deemed essential for a spacecraft to be classified as a space robot, namely, locomotion and autonomy.” (Gao and Chien, 2017) Locomotion or mobility is required for a space robot to conduct the desired operation, like gripping and sample collecting. In addition, it is expected that a space robot will have autonomy at some level. For example, at the most basic level, a space robot will work as a human proxy in space with direct human control. These capabilities, developed through decades of robotic missions, are now being adapted and enhanced to meet the challenges of OOS. This survey will discuss the history of Space Robotics in the past, present, and future.

### 2.1 Past and current space explorations using robots

**FIGURE 2:**
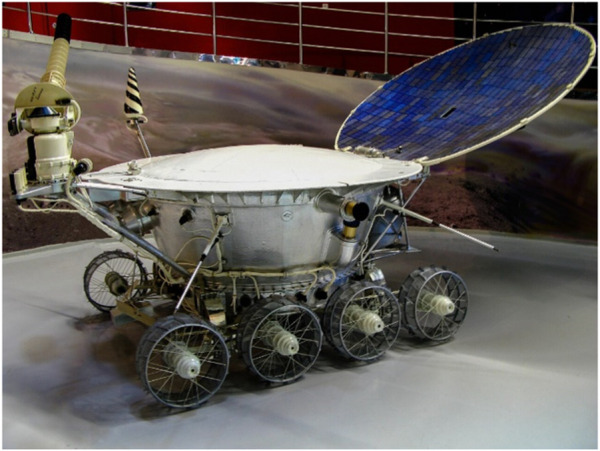
Model of a Soviet Lunokhod program rover. (Credit: Milošević, 2023).

Robotic applications in space exploration have played a crucial role in advancing the understanding of the space environment and exploring the solar system. The initial exploration of Earth’s orbit and the Moon led to the development of cost-effective robotic proxies for space exploration. Various robotic missions with mobility systems like rovers and arms were integral to these efforts. Notable achievements include the successful operation of the first robotic locomotion system on the Moon in 1967 (Surveyor 3), followed by the first planetary robotic arm-mounted drill in 1970 (Luna 16) and the deployment of the first planetary rover, “Lunokhod 1” (Figure 2), in the same year. These milestones, achieved through persistent launch attempts, resulted in significant mission successes and scientific discoveries.

As of 2017, successful missions and robots in Earth’s orbit, the Moon, Mars, and small celestial bodies highlight the pivotal role of robotic arms in orbital mobility and the widespread use of wheeled rovers and stationary landers with robotic arms in planetary exploration. NASA’s Mars rover missions, including Mars Pathfinder (MPF), Mars Exploration Rovers (MERs), and Mars Science Laboratory (MSL), have significantly advanced scientific understanding. Despite its small size, the MPF rover Sojourner made crucial geological discoveries. Larger MER rovers carried advanced science payloads and highlighted impressive mobility, with the Opportunity rover covering over 44 km in more than 4,700 Martian days by 2017. These missions contributed substantially to geological and atmospheric sciences on Mars.

The largest among the three, the MSL rover Curiosity features next-generation instruments for the study of Mars’ geology, atmosphere, environmental conditions, and potential biosignatures. Curiosity utilizes its robotic arm for close-in measurements, including the use of Mars Hand Lens Imager, Alpha Particle X-ray Spectrometer, and sample acquisition analysis. These robotic advancements not only expanded our understanding of other celestial bodies but also provided critical technologies and lessons that are now being applied in OOS to enhance the precision and autonomy of space manipulators.

In 2005, the Japanese Hayabusa robotic mission conducted a comprehensive study of the near-Earth asteroid Itokawa and successfully returned samples to Earth in 2010. This mission garnered significant attention, resulting in special issues in the journal Science, which focused on Itokawa and the analysis of the returned samples. Following this, the Hayabusa-2 mission was launched in 2014 to the asteroid Ryugu, where it successfully collected samples and returned them to Earth in 2020, providing further valuable insights into the composition of primitive celestial bodies.

Another noteworthy project was the Rosetta mission conducted by the European Space Agency (ESA), which was the first mission to rendezvous with a comet, follow it on its orbit around the Sun, and deploy a lander to its surface. The Rosetta lander, named Philae, was equipped with remote sensing and *in situ* instruments for compositional and gas analysis (e.g., Cometary Sampling and Composition and Ptolemy), drilling and sample retrieval (i.e., SD2), and surface measurement (e.g., Surface Electrical Sounding and Acoustic Monitoring Experiment). Unfortunately, the lander’s bounce upon landing and its subsequent tilted resting position limited the application of its arm, sampler, and drill, impacting its measurements and operational lifespan. Despite these challenges, Philae achieved numerous scientific milestones, including discovering organic molecules in the nucleus of 67P/Churyumov-Gerasimenko. These missions underscore the importance of robotic systems in performing complex, high-precision tasks in space—an ability that is directly transferred to OOS missions, where similar technologies are used to maintain and extend the life of existing space assets. A list of successful space robotic missions is presented in Table 1.

**TABLE 1:** Successful space robotic missions as of 2023 (Gao and Chien, 2017).

Launch year	Mission name	Country	Place of operation
1967	Surveyor 3	United States	Moon
1970/1972/1976	Luna 16/20/24	Soviet Union	Moon
1970/1973	Luna 17/21	Soviet Union	Moon
1975	Viking	United States	Mars
1981/2001/2008	Canadarm1/2/Dextre	Canada	ISS
1993	Rotex	Germany	Earth’s Orbit
1996	MPF	United States	Mars
1997	ETS-VII	Japan	Earth’s Orbit
2003	Hayabusa	Japan	Asteroid
2003	MERs	United States	Mars
2004	ROKVISS	Germany	ISS
2004	Orbital Express	United States	Earth’s Orbit
2007	JEMRMS	Japan	ISS
2008	Phoenix	United States	Mars
2008	Robonaut	United States	ISS
2011	MSL	United States	Mars
2012	Chang’E 3	China	Moon
2013	Rosetta	Europe	Comet
2014	Hayabusa-2	Japan	Asteroid
2016	Aolong-1	China	Earth’s Orbit
2018	Chang’E 4	China	Moon
2019	Yutu-2	China	Moon
2018	InSight	United States	Mars
2020	Perseverance	United States	Mars
2020	Ingenuity (Figure 3)	United States	Mars
2020	Zhurong	China	Mars
2021	CMM (Core Module Manipulator)	China	Earth’s Orbit
2022	EMM (Experimental Module Manipulator)	China	Earth’s Orbit
2023	Pragyan (Chandrayaan-3)	India	Moon
2023	SLIM	Japan	Moon

**FIGURE 3:**
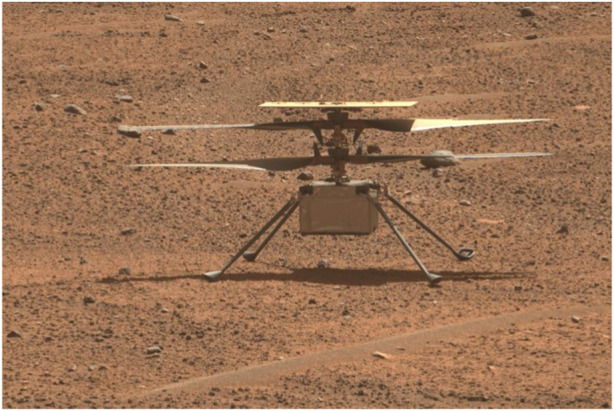
Ingenuity: an autonomous NASA helicopter operated on Mars from 2021 to 2024 (Jet Propulsion Laboratory, 2023).

Beyond landers, robotic manipulators, also known as arms, have played a crucial role in space exploration. The Canadarm series (Canadarm1 and Canadarm2) on the Space Shuttle and the International Space Station serve as prime examples, facilitating various tasks like satellite deployment, retrieval, and on-orbit maintenance. China’s Tiangong space station is also expected to utilize robotic arms (CMM and EMM) for similar functions. The European Space Agency’s Rosetta mission employed a robotic arm on the Philae lander, though operational limitations hampered its full potential. These advancements in robotic manipulators highlight their versatility and growing importance in space exploration endeavors. This versatility is now being harnessed and expanded in OOS missions, where the ability to perform diverse tasks autonomously is increasingly critical. A comparison of some key features of some of these arms is provided in Table 2.

**TABLE 2:** Key specifications of Candarm1/2 and Chinese Space Stations’ robotic arms (Canadian Space Agency, 2024a; Ellery, 2019; Liu, 2014).

Model	Deployed In	DOF	Payload (kg)	Position accuracy (mm)	Orientation accuracy (degree)	End effector velocity, Unloaded (m/s)	End effector velocity, Loaded (m/s)
Canadarm1	US Space Shuttle	6	30,000	50	1	0.6	0.06
Canadarm2	ISS	7	116,000	65	0.7	0.4	0.01
CMM (Core Module Manipulator)	CSS	7	25,000	45	1	0.3	0.02
EMM (Experimental Module Manipulator)	CSS	7	3,000	10	1	0.2	0.03

### 2.2 Future space explorations using robots

From 2025 to 2035, various on-orbit applications will necessitate advanced robotics capabilities, with potential mission operators ranging from space administrations and national governments to private businesses. Envisioned mission objectives encompass a wide range, including space debris removal, rescue operations, planned orbit elevation, inspection, support for deployment, deployment and assembly assistance, repair, refueling, orbit maintenance, mission evolution and adaptation, lifetime extension, and re- and deorbiting. These future missions represent the next frontier in OOS, where robotics will not only perform maintenance but also construct and adapt space infrastructure in real time.

**FIGURE 4:**
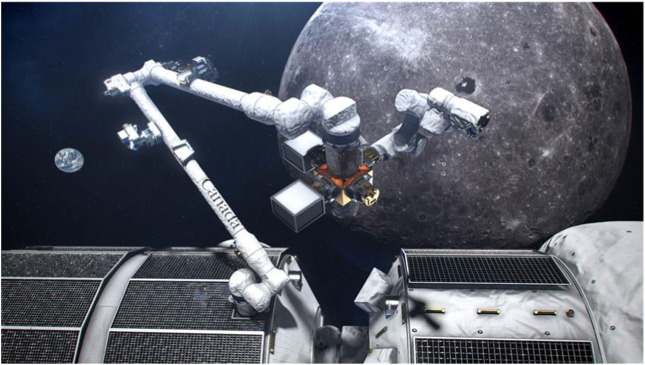
A conceptual illustration of Canadarm3’s robotic arm aboard the Lunar Gateway. (Canadian Space Agency, 2024).

The International Space Station (ISS) remains a valuable platform for scientific experiments in the unique environment of space. Simultaneously, China is actively advancing its space station program, which is expected to be established over this decade, providing a novel space platform for robotic solutions. Additionally, NASA is partnering with CSA, ESA, JAXA, and MBRSC to establish a space station in lunar orbit called “Lunar Gateway”, which is planned to include a science laboratory, a testbed for new technologies, a rendezvous location for exploration of the surface of the Moon, a mission control center for operations on the Moon, and eventually, a stepping stone for voyages to Mars (Canadian Space Agency, 2024b). Lunar Gateway is also host of Canadarm3 (Figure 4); This robotic system will employ advanced software to autonomously carry out certain tasks on the Moon without the need for human involvement (Canadian Space Agency, 2024). These developments are poised to extend the capabilities of OOS by providing new platforms and technologies for autonomous operations in increasingly distant and challenging environments. These orbital robotic missions support scientific exploration both directly and indirectly from Earth’s orbit.

**FIGURE 5:**
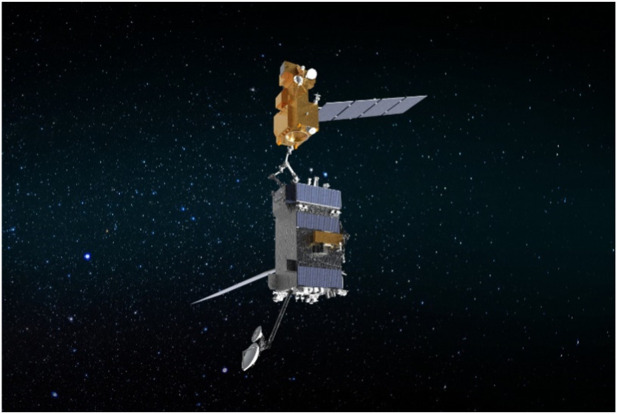
An artistic depiction of OSAM-1 docking with a satellite. (Landsat Science, 2022).

Adding to the future landscape of on-orbit servicing, NASA’s On-orbit Servicing, Assembly, and Manufacturing 1 (OSAM-1) mission, set to launch no earlier than 2025, represents a significant leap forward in robotic servicing technologies. OSAM-1 (Figure 5) will be the first mission to robotically refuel a satellite not originally designed for servicing and will also demonstrate advanced in-space assembly and manufacturing capabilities. This mission recently passed its mission critical design review (CDR), confirming that all elements, including the spacecraft bus, servicing payload, and the Space Infrastructure Dexterous Robot (SPIDER) payload, are ready for the next phase of flight manufacturing, assembly, and integration. OSAM-1 will use a robotic arm and specialized tools to grapple Landsat 7, providing the Earth-observing satellite with a refueling service, followed by the construction of a functional communications antenna and a spacecraft beam using in-space manufacturing techniques. This mission not only showcases the potential of robotic servicing to extend the life of existing satellites but also highlights the future possibilities of constructing and maintaining space infrastructure directly in orbit. This mission not only demonstrates the potential for extending the life of satellites through robotic intervention but also showcases how OOS technologies are evolving to include in-orbit construction, potentially revolutionizing the way space infrastructure is built and maintained. The successful execution of OSAM-1 could pave the way for a more sustainable and versatile approach to space exploration and satellite management in the coming decades (Steigerwald, 2022).

“Space exploration and exploitation depend on tasks such as inspecting, refueling, upgrading, repairing, or rescuing satellites, removing orbital debris, and construction and maintenance of large orbital assets and infrastructures” (Papadopoulos et al., 2021). In the past, servicing tasks in Low Earth Orbit (LEO) were primarily conducted through astronaut extravehicular activities (EVAs), but these operations are risky, costly, and time-consuming. For critical assets in high-altitude orbits like Geosynchronous Orbits (GEO), EVAs are not viable. This shift towards robotic on-orbit servicing (OOS) represents a significant change in how these essential tasks are performed, utilizing space manipulator systems (SMSs) that feature satellite bases with robotic arms and vision systems.

Since the 1990s, research on SMSs has grown due to their potential for repairing, rescuing, and refueling satellites, and removing space debris. The increasing population of space debris heightens the risk of collisions, making robotic servicing missions crucial for safely capturing targets with robotic arms, given operational constraints. These developments underline the importance of continuing to advance OOS technologies to ensure the sustainability and safety of space operations as the space environment becomes more congested.

In addition to large-scale robotic systems like Canadarm, ongoing research focuses on integrating small robotic mechanisms into CubeSats for active space debris removal. These small satellites, often designed to meet CubeSat standards, are equipped with robotic manipulators intended to chase, capture, and de-orbit non-cooperative debris in Low Earth Orbit (LEO). For instance, debris chaser satellites can track nearby debris, perform low-thrust rendezvous, and use their manipulators to grasp and stabilize the debris before de-orbiting it to ensure safe atmospheric burn-up. These smaller systems complement larger OOS efforts by providing scalable solutions that can address specific challenges, such as debris removal, in a cost-effective manner. Innovations such as the REMORA CubeSat (McCormick et al., 2018), equipped with miniature robotic arms and end-effectors for debris attachment, demonstrate the potential of CubeSat-based systems to mitigate collision risks from large debris objects. These cost-effective, scalable solutions complement larger robotic systems and enhance overall space debris mitigation efforts (Hakima et al., 2018; McCormick et al., 2018; Nishida and Kawamoto, 2011; Reintsema et al., 2010; Sah et al., 2022).

Moreover, the on-orbit servicing ecosystem has seen the emergence of several innovative companies and start-ups that are actively contributing to the development and deployment of advanced OOS technologies. For example, French startup Infinite Orbits, founded in 2017 and based in Toulouse, specializes in life and mission extension services for satellites in geostationary orbits (GEO). They utilize dedicated satellite servicers equipped with optical sensors for target-agnostic guidance, navigation, control (GNC), and docking systems, enabling them to extend the operational life of GEO satellites by up to 5 years. Another player, Space Machines Company from Adelaide, Australia, founded in 2018, focuses on in-orbit assembly of large space structures using its space mobility platform, Optimus, which supports a range of services including refueling, satellite repairs, and lifetime extensions. In the U.S., Scout Aerospace, founded in 2017 in Atlanta, constructs orbital transfer vehicles (OTVs) for precise payload deployment and servicing in orbit. Their AstroLabe platform facilitates easier repairs and upgrades for multiple payloads. Lastly, Russian startup Orbital Express, founded in 2020, builds space tugs for small satellite orbital positioning and interplanetary missions, offering services such as orbit phasing, deorbiting, and life extension. These companies exemplify the growing industrial interest in OOS and highlight the diverse capabilities being developed to support the future of space operations, reflecting the increasing commercialization and innovation in this critical area (Infinite Orbits, 2024; Orbital Express, 2024; Scout Aerospace LLC, 2024; Space Machine Company, 2024).

## 3 On-orbit servicing - An overview

Before delving into the intricacies of each On-Orbit Servicing (OOS) subsystem, it is essential to establish a foundational understanding of the general tasks, system-level requirements, and overall composition of OOS robots. By doing so, we can better appreciate how these subsystems work together to enable the execution of complex tasks in the challenging environment of space.

OOS robots are designed to perform a variety of critical tasks in space, including inspection, repair, refueling, assembly, and debris removal. Each of these tasks demands a unique combination of precision, dexterity, and reliability, which are achieved through the careful integration of multiple subsystems. The following subsections outline these tasks, the system-level requirements that must be met to perform them effectively, and the general composition of OOS robotic systems.

• OOS Tasks: OOS robots perform a variety of critical tasks in space, including:
  o Inspection: Meticulously examining spacecraft for damage or malfunction.
  o Repair: Fixing or replacing faulty components on spacecraft
  o Refueling: Replenishing a spacecraft’s propellant for extended operation.
  o Assembly: Constructing large structures in space from prefabricated modules.
  o Debris Removal: Deorbiting or safely disposing of defunct satellites and space debris.

These tasks require not only advanced robotic technology but also a deep understanding of the space environment and the specific challenges associated with operating in microgravity. The successful execution of these tasks is crucial for the sustainability of space operations, as they enable the maintenance and extension of spacecraft lifespans, the construction of new space infrastructure, and the mitigation of space debris.

• System-Level Requirements: OOS robotic systems must be designed to meet several demanding requirements:
  o Precision and Dexterity: OOS robots need exceptional precision and dexterity to handle delicate tasks in microgravity.
  o Safety and Reliability: The safety of the OOS robot and the target spacecraft is paramount.
  o Autonomy: OOS robots may need to operate with some level of autonomy, especially during communication delays with Earth.
  o Efficiency: OOS operations should be completed efficiently to minimize mission costs.

Meeting these requirements is essential for the success of OOS missions, as even minor errors can have significant consequences in the unforgiving environment of space. Therefore, each subsystem must be meticulously designed and rigorously tested to ensure it can perform reliably under these challenging conditions.

**FIGURE 6:**
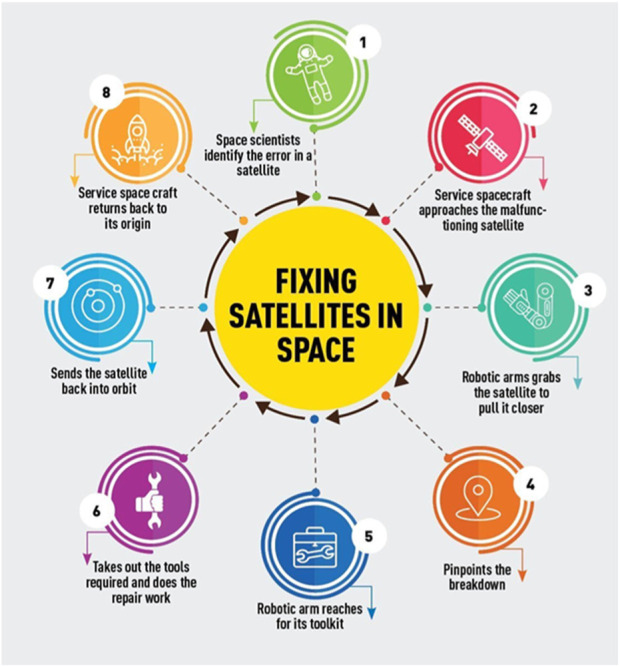
On-orbit service or repair process (Choudhary, 2018).

• General System Composition: An OOS robotic system typically comprises several key subsystems (Figure 6):
  o Arm: A robotic arm with multiple joints enables reaching, grasping, and manipulating objects in space.
  o Sensors and Cameras: Vision systems and various sensors provide crucial data about the surrounding environment and the target spacecraft.
  o Toolkits: OOS robots carry an array of specialized tools for various repair and maintenance tasks.
  o Computer Systems and Software: Onboard computers and software process sensor data, control robot movements, and execute mission plans.
  o Power Source: A reliable power source ensures the robot can function effectively throughout the mission.

These subsystems work in concert to provide OOS robots with the capabilities they need to perform complex tasks autonomously or semi-autonomously. Understanding how these subsystems interact and complement each other is key to appreciating the full capabilities of OOS technologies.

By understanding these general aspects of OOS, we can better appreciate the complexities involved and the critical role each subsystem plays in enabling these remarkable robots to service spacecraft in the harsh space environment.

### 3.1 Ground test facilities for on-orbit servicing

**FIGURE 7:**
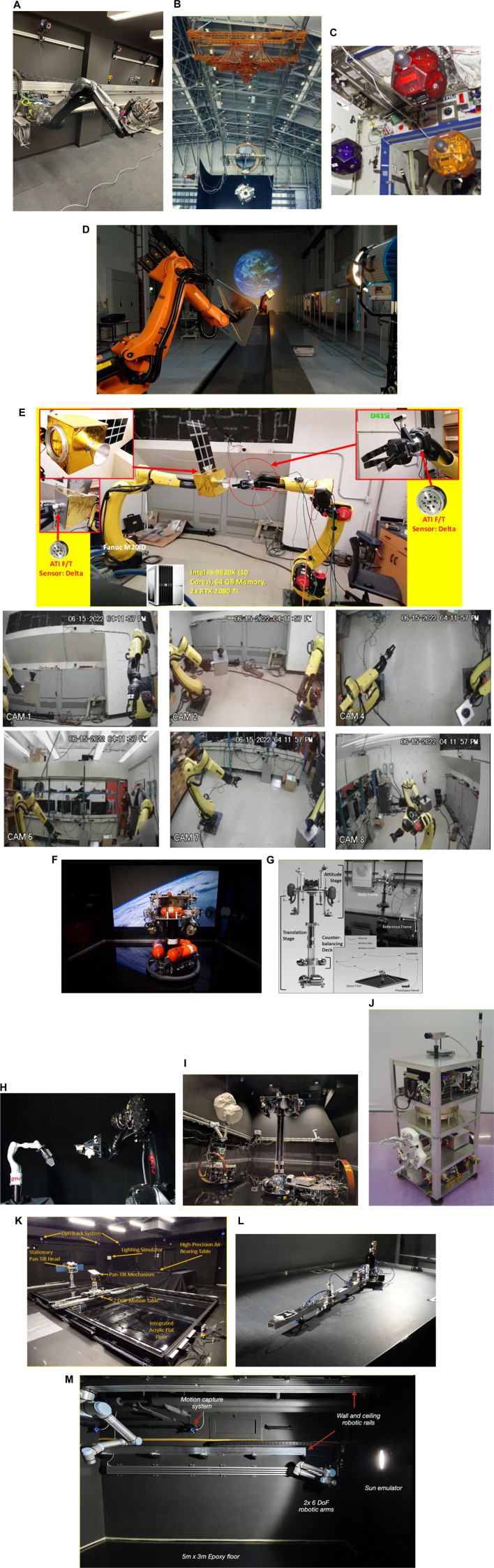
**(A)** Illustration of GIMLI mounted on a KUKA robotic arm. (ESA, 2024). **(B)** Image o f NASA Rendezvous Docking Simulator. (NASA, 2024) **(C)** Image of SPHERES aboard ISS (MIT Space Systems Laboratory, 2024). **(D)** A sample simulation scenario featuring Earth in the background. (DLR, 2024). **(E)** 6DOF Hardware-in-The-Loop Testbed for Autonomous Robotic OOS (Al Ali and Zhu, 2023). **(F)** Autonomous Spacecraft Testing of Robotic Operations in Space (ASTROS) (Dynamic and Control Systems Laboratory, 2024). **(G)** ADAMUS testbed (Saulnier et al., 2014). **(H)** GMV’s platform-art^©^ advanced robotics laboratory (GMV, 2022). **(I)** Impage of 6-DOF spacecraft simulators at Caltech’s Aerospace Robotics and Control Laboratory (Nakka et al., 2018). **(J)** AUDASS II vehicle (Tracy, 2005). **(K)** Maneuver kinematics and dynamics testbed (Florida Tech, 2024). **(L)** Planar air-bearing microgravity simulator (Rybus et al., 2013). **(M)** ZeroG lab facility at University of Luxembourg (Muralidharan et al., 2022).

A critical aspect of preparing On-Orbit Servicing (OOS) robots for space missions involves rigorous ground testing in facilities specifically designed to emulate on-orbit scenarios. These facilities allow for experimentation with proximity operations, which are crucial for tasks such as docking, capture, and manipulation of spacecraft in space. Several leading facilities are equipped to simulate the unique conditions and challenges of on-orbit operations:

• ESTEC’s ORBIT Facility: The Orbital Robotics Bench for Integrated Technology (ORBIT) at the European Space Agency’s ESTEC is a premier facility within the Orbital Robotics Lab. This state-of-the-art setup features a flat floor environment that simulates microgravity by providing frictionless motion in three degrees of freedom (2D translation and 1D rotation). It supports a variety of OOS experiments, including those related to Active Debris Removal and satellite servicing. The lab’s MANTIS (MANeuverable Testbed for In-orbit Simulation) and REACSA (REcap + ACrobat + SAtsim) platforms enable the simulation of orbiting systems and interactions between free-floating bodies. Additionally, the GIMLI (Gripping Interface for Manipulation, Locking, and Interaction) system facilitates docking operations, allowing for the study of compliant connections between robotic systems. These capabilities make ORBIT an invaluable resource for testing and refining proximity operations in OOS missions (Figure 7A) (ESA, 2024). The ability to simulate microgravity and proximity operations in a controlled environment is critical for the development of reliable and safe OOS systems. These tests ensure that the robotic systems will function as expected when deployed in space, where errors can be costly and difficult to rectify.
• NASA’s Proximity Maneuver Simulators: NASA has developed advanced proximity maneuver simulators, starting with the Rendezvous Docking Simulator (RDS) at Langley Research Center, originally designed for docking simulations between Gemini and Agena spacecraft, and later retrofitted for Apollo missions. This large-scale facility (Figure 7B) featured a 65 m × 4.6 m × 12.2 m motion envelope, with the chaser vehicle mounted in a 3 DOF gimbal frame, suspended by cables. Today, NASA operates some of the largest air-bearing dynamics simulation systems, including the 21.3 m × 29.9 m Air Bearing Floor (ABF) at Johnson Space Center and the 13.4 m × 26.2 m floor at the Flight Robotics Laboratory (FRL) at Marshall Space Flight Center, which are used for testing rendezvous and docking maneuvers. (Wilde et al., 2019).

These facilities play a crucial role in validating the proximity maneuvering capabilities of OOS robots, ensuring that they can execute docking procedures with precision and reliability, even in the challenging environment of space.

• SPHERES at MIT Space Systems Laboratory: The Synchronized Position Hold Engage and Reorient Experimental Satellite (SPHERES) system, developed by the MIT Space Systems Laboratory in collaboration with NASA, DARPA, and Aurora Flight Sciences, is a pioneering facility designed to test sensor, control, and autonomy technologies for satellites in a zero-gravity environment. Operating aboard the International Space Station (ISS), the SPHERES system consists of small satellites capable of precise rotation and translation in all directions, controlled by twelve carbon dioxide thrusters. These satellites utilize ultrasound beacons and receivers to determine their relative positions, enabling the testing of formation flight technologies and other critical satellite functions. With over one hundred test sessions conducted, current research using SPHERES includes advanced investigations into factor graph-based simultaneous localization and mapping (SLAM) and the development of algorithms for real-time planning and parameter estimation under uncertainty. This facility plays a crucial role in advancing the capabilities of autonomous satellite systems (Figure 7C) (MIT Space Systems Laboratory, 2024).
• DLR’s European Proximity Operations Simulator (EPOS 2.0): Located at the DLR Space Operations and Astronaut Training in Oberpfaffenhofen, EPOS 2.0 is a state-of-the-art facility (Figure 7D) designed for the simulation of space-based inspection and approach maneuvers, essential for rendezvous operations. This large-scale facility is crucial for missions involving orbital maintenance and towing services, where complex rendezvous and docking procedures must be extensively tested and verified. EPOS 2.0 features two six-degree-of-freedom industrial robots, one mounted on a 25-m-long rail, enabling real-time simulations of satellite approaches. The facility is notable for its sub-millimeter positioning accuracy over 25 m and its high command rate of 250 Hz. Additionally, a high-performance solar simulator provides realistic ambient lighting, which is critical for testing optical sensors. EPOS 2.0 is instrumental in developing and validating navigation and docking procedures, particularly for non-cooperative, tumbling satellites, and is at the forefront of research into robotic systems for the deorbiting of space debris (DLR, 2024). EPOS 2.0’s capabilities are vital for advancing OOS technologies, particularly in scenarios involving non-cooperative targets, where precise maneuvering and docking are required to successfully complete servicing missions.
• 6DOF Hardware-in-The-Loop Testbed, York University: Located at Space Engineering Lab at York University, the hardware-in-the-loop ground testbed featuring active gravity compensation via software-in-the-loop integration, specially designed to support research in autonomous robotic OOS. It is is designed to accurately simulate the dynamic behaviors of free-floating robotic manipulators and spacecraft under microgravity conditions. The testbed comprises two 6DOF robotic manipulators, one 3-finger gripper, and sensors like cameras, force/torque sensors, and tactile tensors (Figure 7E). It can test and validate technologies related to autonomous tracking, capture, and post-capture stabilization in OOS.
• ASTROS, Georgia Tech Aerospace Engineering: The Autonomous Spacecraft Testing of Robotic Operations in Space (ASTROS) facility at Georgia Tech is a state-of-the-art 5-DoF spacecraft simulator and frictionless motion experimental platform. This facility is designed to validate and evaluate guidance, navigation, and control (GNC) algorithms for in-space rendezvous using dedicated hardware. The test arena features a 4 m × 4 m flat epoxy floor where experimental platforms can hover without friction, simulating space-like conditions. The ASTROS platform is equipped with advanced sensing, computation, and actuation systems, including air thrusters and variable-speed control moment gyros (VSCMGs), allowing for precise control of spacecraft motion during testing. This facility is critical for developing autonomous proximity operations, which are essential for on-demand on-orbit servicing and refueling of space assets (Figure 7F) (Dynamic and Control Systems Laboratory, 2024).
• ADAMUS, University of Florida: The ADvanced Autonomous MUltiple Spacecraft laboratory (ADAMUS) at the University of Florida is a cutting-edge facility featuring a six-degree-of-freedom hardware-in-the-loop simulator designed for small spacecraft. This testbed allows for the testing and validation of novel guidance, navigation, and control (GNC) algorithms in a ground-based environment that closely mimics the conditions of space. Unlike many other simulators that rely on simulated dynamics and servo actuators, the ADAMUS testbed controls all degrees of freedom using real thrusters, providing a highly realistic simulation of spacecraft behavior. The facility is particularly well-suited for rapid prototyping and experimental validation of GNC methodologies, significantly reducing the reliance on lengthy numerical simulations. The unique design of the ADAMUS platform also includes a matched variable-mass counterbalance system to simulate near-gravity-free motion, further enhancing the accuracy of the tests conducted at this facility (Figure 7G) (Saulnier et al., 2014).
• Platform-art, GMV: Platform-art^©^, located at GMV’s Madrid office, is an advanced robotic testbed designed for the testing and validation of space mission systems. This 20 × 6 × 5 m facility is unique in Europe and incorporates state-of-the-art mobile robotics technology. It is a key resource for the development of guidance, navigation, and control (GNC) systems for a wide range of space missions, including space debris capture, formation flying, and planetary exploration. The facility provides thorough ground validation, emulating space conditions to ensure that GNC systems perform reliably during actual missions, where in-flight testing is often impractical due to cost and limited flight opportunities (Figure 7H) (GMV, 2013).
• Spacecraft Dynamics Simulator Facility, Caltech Aerospace Robotics and Control Lab: The Spacecraft Dynamics Simulator Facility at Caltech’s Aerospace Robotics and Control Lab is equipped with a high-precision epoxy flat floor, air-bearing systems, and the M-STAR platform to simulate spacecraft dynamics with full six degrees of freedom. The facility includes an industrial air compressor and high-pressure storage tanks to supply air to the flat air bearings and thrusters on the simulator. The pose of the spacecraft is tracked using a VICON Motion Capture system, enabling precise control and simulation of spacecraft maneuvers. This facility is essential for testing CubeSat dynamics and control algorithms in a controlled environment that closely mimics the conditions of space (Figure 7I) (Nakka et al., 2018).
• AUDASS, US Naval Postgraduate School: The Autonomous Docking and Spacecraft Servicing Simulator (AUDASS) at the US Naval Postgraduate School is designed for on-the-ground testing of satellite servicing and proximity formation flight technologies. The simulator comprises two independent robotic vehicles, a chaser, and a target, which float on a polished granite table via air pads, providing a frictionless support for simulating zero-g dynamics in two dimensions. This facility allows for the testing of autonomous rendezvous and docking procedures, with a particular focus on fluid transfer between spacecraft. AUDASS is an invaluable tool for developing and refining the technologies required for satellite servicing missions (Figure 7J) (Naval Postgraduate School, 2024; Tracy, 2005).
• ORION, Florida Institute of Technology: The Orbital Robotics Interaction On-Orbit Servicing and Navigation (ORION) Laboratory at Florida Institute of Technology features a unique combination of a Cartesian robot and an air-bearing flat-floor system. The facility includes a 5.94 m × 3.60 m integrated flat floor and a high-precision air-bearing table, allowing for the study of the dynamics and kinematics of relative motion and contact dynamics of space vehicles. This setup is ideal for simulating and analyzing the complex interactions involved in on-orbit servicing and navigation, contributing significantly to the advancement of space robotics technologies (Figure 7K) (Florida Tech, 2024; Wilde et al., 2019).
• Space Research Center, Polish Academy of Sciences (PAS): The Space Research Centre of the Polish Academy of Sciences (PAS) operates a planar air-bearing microgravity simulator designed for the verification of space robotics numerical simulations and control algorithms. This facility features a 2 × 3 meter granite table with air bearings that provide negligible friction, allowing free planar motion of satellite-manipulator systems. Each manipulator link is independently supported, enabling the testing of long and heavy manipulators that significantly influence the base position and orientation. This simulator is crucial for developing control and trajectory planning methods for free-floating systems, making it a key resource for advancing space robotics research in Poland (Figure 7L) (Rybus et al., 2013).
• Zero-G Lab, University of Luxembourg: The Zero-G Lab at the University of Luxembourg’s Interdisciplinary Space Master (ISM) program is designed to simulate a microgravity environment for testing the movement of in-orbit robotics, satellites, and other spacecraft. This facility functions similarly to an air hockey platform, allowing researchers and students to study how spacecraft and orbital robotics can be controlled in a microgravity environment. The lab provides a unique opportunity to understand and forecast the behavior of space systems in the absence of gravity, which is essential for successful in-orbit operations (Figure 7M) (Olivares-Mendez and Aouada, 2024)

These ground test facilities are indispensable for the ongoing development and refinement of OOS technologies. They provide a controlled environment where critical subsystems and operations can be thoroughly tested and validated, ensuring that when OOS robots are deployed in space, they are capable of performing their tasks with the required precision and reliability.

For more detailed information on these and other test facilities, see (Wilde et al., 2019).

## 4 Sensing of pose and state

Accurate and fault-tolerant navigation systems are among the most critical components of future on-orbit servicing missions. The ability to precisely determine the pose and state of objects in space is essential for tasks such as docking, capturing, and repairing spacecraft. Failure to provide reliable pose and state sensing could result in catastrophic failure or damage to neighboring space objects, potentially jeopardizing entire missions. For instance, reliable 6-DOF (Degrees of Freedom) pose information is crucial when approaching and docking with the International Space Station (ISS). As we explore the various methods and tools used in pose and state sensing, we will see how advancements in this area directly contribute to the safety and efficiency of OOS missions.

A summary of methods and tools found in this section can be found in Table 3.

**TABLE 3:** A Summary of methods and tools in sensing of pose and state.

Method/Tool	Description	Pros	Cons
Radar or Altimeter (Kriegsman, 1966)	Removes bias measurement errors in IMU using least squares estimation	Improves accuracy of IMU data	May require additional hardware
Vision-based method with ICP (Iterative Closest Point) (Besl and McKay, 1992)	Finds all 6-DOF pose information of an object	Can be used for complex object shapes	Can be computationally expensive
Approximate Nearest Neighbor (ANN) with ICP (Greenspan and Yurick, 2003)	Improves ICP convergence speed for 3D registration	Reduces processing time	May not improve accuracy significantly
Differential carrier phase GPS (Wolfe and Speyer, 2004)	Estimates relative positions of satellites in Low Earth Orbit (LEO)	Provides high accuracy for relative positioning	Requires specialized GPS receivers Only works in LEO
Rendezvous Laser Radar (RVR) (Mokuno et al., 2004)	Effective for navigation during the final approach phase of docking	High accuracy for short-range measurements	May be expensive
Laser Camera System (LCS) (Samson et al., 2004)	Estimates pose of an object accurately and is immune to dynamic lighting conditions	Robust to variations in lighting	May require complex calibration procedures
Range data-based motion estimation (Hillenbrand and Lampariello, 2005)	Estimates the motion of free-floating objects	May not require specialized cameras	Accuracy may be limited by the range of data quality
LIDAR sensor (Lu and Tomizuka, 2006)	Used as a vehicle detection system	Can provide high-resolution 3D data	May be expensive
Kalman filter with laser-vision data (Aghili and Parsa, 2007)	Estimates motion and predicts the trajectory of satellites	Good at filtering out noise and predicting future states	Performance depends on the quality of the input data
Adaptive vision system (Aghili and Parsa, 2008)	Used for guidance during satellite interception and capture	Can handle variations in target appearance	May require significant computational resources
3D camera with Kalman filter and ICP (Aghili et al., 2011)	Estimates pose of space objects	Combines the strengths of different techniques for improved accuracy and robustness	May be more complex to implement
X-ray pulsars (Liu et al., 2015)	Novel method for relative navigation between spaceships using pulsars	Provides long-range navigation capability	Requires specialized equipment to detect X-ray pulsars
Probabilistic motion modeling (Tweddle et al., 2015)	Creates a 3D map with position, orientation, and motion details of a spinning object	Can handle complex object dynamics	Computational demands may increase with map size
Optical sensor data with least squares (Benninghoff and Boge, 2015)	Estimates center of mass and moments of inertia of a non-cooperative satellite	Critical for safe maneuvering during docking	May not be effective for all lighting conditions
Laser scanner and IMU with ICP and Kalman filter (Aghili and Su, 2016)	Provides robust relative navigation	Combines the strengths of different sensors and filters for high accuracy	Increased complexity due to multiple sensors and algorithms
Camera-based tracking (Nassir Workicho Oumer, 2016)	Offers a cheaper alternative to LiDAR for close-range satellite servicing	Less expensive and lighter weight	Performance can be affected by lighting conditions

### 4.1 Traditional techniques

Early solutions for determining an object’s pose and state relied on well-established techniques, which laid the groundwork for more advanced methods used in OOS today. One approach involved using radar or altimeter data to remove bias errors in Inertial Measurement Unit (IMU) readings through least squares estimation (Kriegsman, 1966). Vision-based methods, such as the Iterative Closest Point (ICP) algorithm, were also introduced to obtain complete 6-DOF pose information (Besl and McKay, 1992). These techniques, though foundational, demonstrated the importance of precise pose estimation in the context of space operations, where even minor errors can have significant consequences.

**FIGURE 8:**
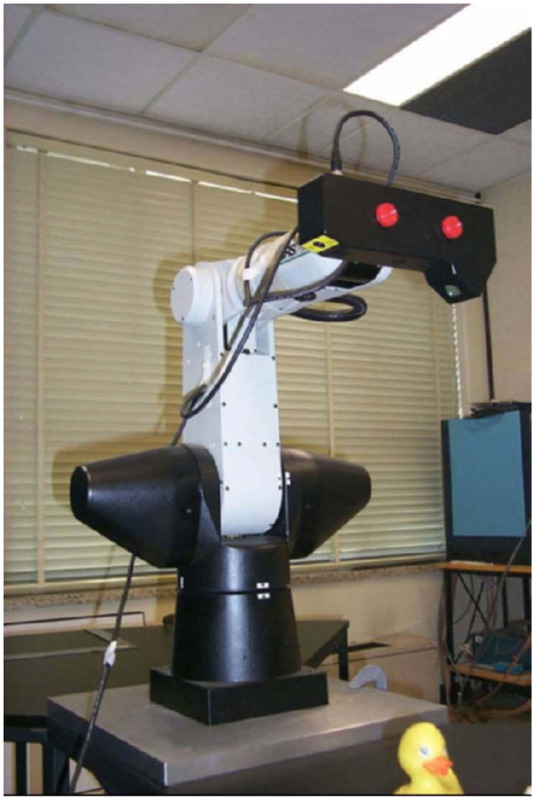
Biris range scanner on the end-effector of a CRS A465 6-DOF articulated manipulator. (Greenspan and Yurick, 2003).

Other traditional methods focused on estimating and predicting rotational motions, particularly for rigid bodies without external forces or moments (Masutani et al., 1994). To improve the convergence speed of ICP for 3D registration purposes, the Approximate Nearest Neighbor (ANN) technique was implemented (Greenspan and Yurick, 2003). This approach was used to register range images of four toy objects collected using a Biris range scanner mounted on the end-effector of a CRS A465 6-DOF articulated manipulator, as illustrated in Figure 8. Additionally, differential carrier phase GPS measurements were demonstrated as a method for estimating the relative positions of widely separated satellites in Low Earth Orbit (LEO) (Wolfe and Speyer, 2004). These early techniques established a foundation upon which more advanced, sensor-based methods were developed.

### 4.2 Sensor-based techniques

As sensor technology advanced, new possibilities emerged for more accurate and reliable pose and state sensing in space. These sensor-based techniques have become instrumental in modern OOS missions, where the need for precision and fault tolerance is paramount.

**FIGURE 9:**
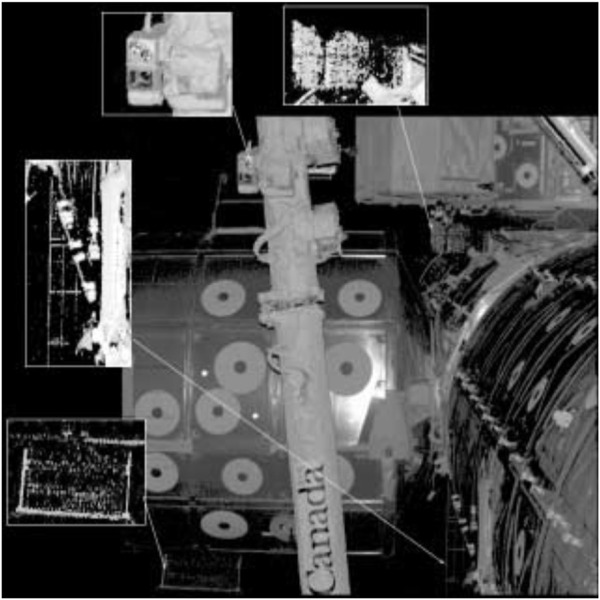
Two-dimensional projection of the three-dimensional high-resolution intensity data acquired by the LCS in orbit (Samson et al., 2004).

For instance, during an experiment conducted by the National Space Development Agency of Japan (NASDA), an uncrewed autonomous rendezvous docking operation was performed using a rendezvous laser radar (RVR) as the primary navigation sensor in the final approach phase. This experiment demonstrated the effectiveness of laser-based sensing for critical docking tasks in space (Mokuno et al., 2004). Similarly, the Laser Camera System (LCS) was introduced as a tool capable of estimating object pose accurately while being immune to dynamic lighting conditions, a significant advantage in the challenging environment of space (Samson et al., 2004). As shown in Figure 9, the LCS successfully captured a two-dimensional projection of three-dimensional high-resolution intensity data while in orbit, highlighting its versatility in various space environments.

These sensor-based advancements are crucial for enhancing the robustness and accuracy of OOS operations, ensuring that spacecraft can perform complex maneuvers with the precision required for successful mission outcomes.

### 4.3 Data fusion techniques

To further improve the accuracy and robustness of pose and state sensing, many modern techniques leverage data fusion, combining information from multiple sensors. This approach is particularly valuable in complex OOS tasks, where relying on a single sensor type may not provide sufficient reliability.

One notable example is the proposed architecture for estimating the dynamic state, geometric shape, and model parameters of objects in orbit using cooperative 3D vision sensors (Lichter and Dubowsky, 2004). This method illustrates the potential of using multiple data streams for comprehensive object characterization, a critical capability in OOS where understanding the target object’s state is vital for successful interaction. Similarly, data from range measurements was used to estimate motion for free-floating objects (Hillenbrand and Lampariello, 2005), and LIDAR sensors were explored as vehicle detection systems, demonstrating their potential for enhancing space situational awareness (Lu and Tomizuka, 2006).

**FIGURE 10:**
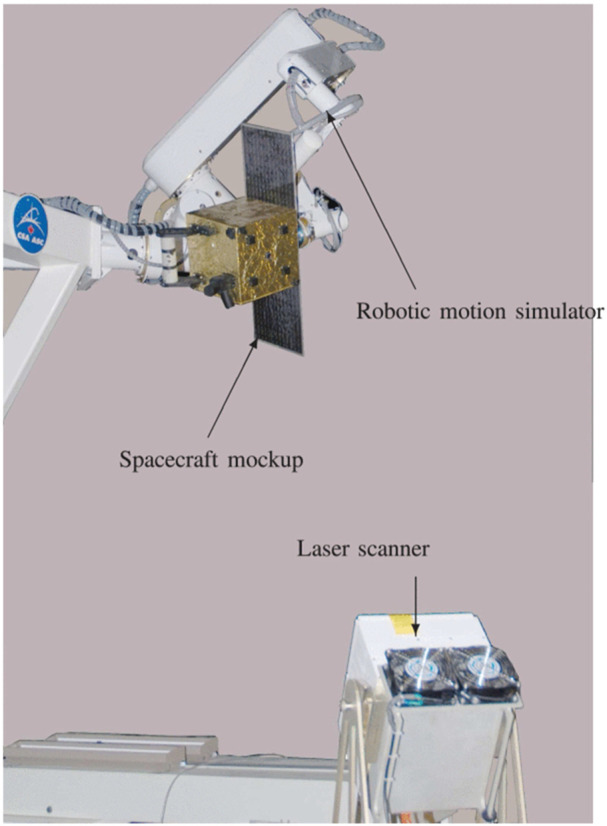
A Neptec laser rangefinder scanner captures the pose of a satellite mockup, operated by a manipulator arm controlled through a simulator based on orbital dynamics. (Aghili and Su, 2016).

Data fusion is especially useful when dealing with dynamic and unpredictable scenarios, such as intercepting and capturing non-cooperative satellites. For example, a noise-adaptive Kalman filter combined with laser-vision data was introduced to estimate motion and predict the trajectory of a free-falling tumbling satellite (Aghili and Parsa, 2007). This technique, along with an adaptive vision system proposed as a guidance tool for capturing non-cooperative satellites, showcases the system’s ability to handle variations in target appearance (Aghili and Parsa, 2008). Moreover, combining a 3D camera with a Kalman filter and ICP allowed for accurate pose tracking of space objects even with temporary camera signal loss (Aghili et al., 2011). More recently, a method combining a laser scanner (Figure 10) and IMU with ICP and a Kalman filter demonstrated robust relative navigation, reinforcing the importance of integrating multiple sensors to achieve reliable performance (Aghili and Su, 2016). A robust vision system for robots performing grasping tasks was also proposed (Aghili, 2022). This system is designed to handle temporary vision loss and optimize movements under constraints. The system combines several techniques: 1) image registration to track objects, 2) a Kalman filter to estimate the object’s state, 3) fault detection to identify vision issues, and 4) a path planner to optimize robot movements. Simulations have shown that the system can successfully grasp moving targets even with complete vision loss for short periods.

### 4.4 Advanced techniques

Recent advancements in sensing technology have introduced novel approaches that promise to further enhance the capabilities of OOS robots. These advanced techniques are essential for addressing the increasingly complex challenges faced in modern space missions.

One such technique involves the use of X-ray pulsars for relative navigation between spaceships venturing into deep space. This method leverages the predictable nature of pulsar signals to estimate a spacecraft’s position and velocity, offering a potential solution for deep-space navigation challenges (Emadzadeh and Speyer, 2011). Additionally, Convolutional Neural Networks (CNNs) have emerged as a powerful tool for pose estimation. They have been explored to provide faster and more reliable initial estimates of a target’s orientation during spacecraft docking procedures (Oestreich et al., 2020). Similarly, CNNs have been implemented to directly predict the pose of a spacecraft without requiring a 3D model, showing great promise for tasks involving uncooperative spacecraft (Garcia et al., 2021; Piazza et al., 2022).

These advanced techniques represent the future of pose and state sensing in OOS, where the need for high accuracy, robustness, and real-time processing is more critical than ever.

### 4.5 Cooperative vs non-cooperative targets

The level of cooperation from the target object significantly impacts the choice of sensing methods, making it crucial to tailor approaches based on whether the target is cooperative or non-cooperative. Cooperative targets, which actively transmit information, allow for the use of methods such as cooperative 3D vision systems (Lichter and Dubowsky, 2004). However, non-cooperative targets, which do not provide such information, require sensing techniques that rely solely on the capabilities of the approaching spacecraft.

**FIGURE 11:**
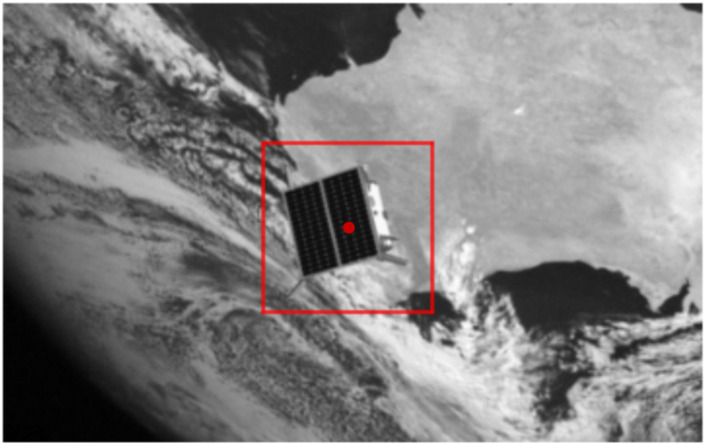
A spacecraft bounding box detection by LSPnet. (Garcia et al., 2021).

For non-cooperative objects, several methods have been explored to overcome the challenges posed by their uncooperative nature:

• Cameras and LIDAR sensors: These sensors capture visual and 3D data of the target, allowing for pose estimation and motion tracking. Camera-based tracking offers a cheaper and lighter alternative to LIDAR for close-range satellite servicing, although its performance may be affected by variations in space lighting conditions (Nassir Workicho Oumer, 2016).
• Estimating object properties: Researchers have developed methods to estimate the properties of non-cooperative satellites, such as mass, center of mass, and inertia, without relying on information from the target itself. One approach involves gently nudging the satellite with a flexible rod while measuring its response using force-moment sensors (Meng et al., 2019).
• Convolutional Neural Networks (CNNs): As previously mentioned, CNNs are being explored for pose estimation of uncooperative spacecraft. These networks can learn from large datasets of images and point clouds to directly predict the pose of a target, even without a pre-defined 3D model (Garcia et al., 2021; Piazza et al., 2022). As demonstrated in Figure 11, CNNs can detect the bounding box of a spacecraft using models like LSPnet, enhancing the accuracy of the pose estimation process.
• Tactile Sensors: Recent advancements highlight the importance of space-qualifiable tactile sensors for tasks like orbital debris characterization and manipulation. These sensors enable non-traditional grasping and manipulation techniques, such as those augmented by microspines or gecko-inspired adhesives. By integrating tactile feedback with control systems, these sensors can improve the accuracy and safety of in-orbit operations, including debris handling and in-situ construction on celestial bodies (Di, 2024; NASA, 2020).

The ongoing development of robust sensing methods tailored to non-cooperative or unknown objects underscores the importance of adaptability and innovation in OOS. These methods are critical for ensuring the success of missions where traditional sensing techniques may not be sufficient.

## 5 Motion planning

**FIGURE 12:**
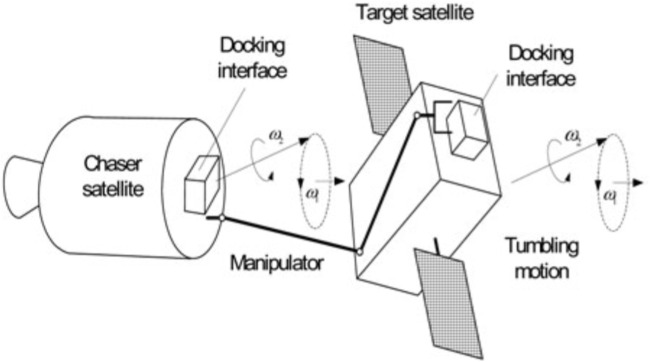
Concept of the chaser satellite captured the tumbling satellite (Ma et al., 2007).

Manipulator motion planning provides trajectory and attitude profiles essential for on-orbit servicing (OOS). As depicted in Figure 12, the chaser satellite successfully captures the tumbling satellite, demonstrating the critical role of precise motion planning in ensuring stable and accurate docking, even when the target satellite is in an uncontrolled tumbling state. This section will explore the unique challenges of motion planning in space and the strategies developed to overcome them, ultimately enabling the safe and efficient operation of OOS missions.

A summary of challenges and their respective solutions in this section can be found in Table 4.

**TABLE 4:** Summary of challenges and their respected solutions.

Challenges	Solutions	Pros	Cons
Unpredictable motion of free-floating robots	Safe zones to avoid unpredictable motion (Papadopoulos, 1992)	Creates safe areas for robot operation	May limit usable workspace
Dynamic singularities limiting workspace	Workspace models considering singularities (Papadopoulos and Dubowsky, 1993)	Improves planning efficiency by avoiding singularities	Requires additional computational power for modeling
Shrinking workspace for robots capturing large objects	Grasping postures for large objects (Papadopoulos, 1993)	Enables manipulation of larger objects	May require complex pre-programmed grasping strategies
Bendy arms causing base vibrations	Separate control systems for precise arm movement and base minimization (Nenchev et al., 1999)	Improves manipulation precision and reduces spacecraft disturbances	Requires more complex control systems
Collision-free movement for multi-joint robots	Planning algorithms with “trees” exploring space (Kuffner and LaValle, 2000)	Efficiently finds collision-free paths	Can be computationally expensive for overly complex environments
Limited control for robots without main engines	Smoother paths are designed directly in joint space (Tortopidis and Papadopoulos, 2007)	Achieves smoother motions without requiring small, jerky movements	May limit flexibility for real-time adjustments during the operation
Uncertainties about object properties	Control methods without precise knowledge of the object (Abiko et al., 2006)	Functions even without perfect object data	May require additional sensors or assumptions about object properties
Capturing and stopping spinning satellites	Reaction null space for controlling robot orientation (Dimitrov and Yoshida, 2004)	Maintains robot stability during the capture of spinning objects	Requires advanced control systems and precise thruster control
Smooth motions for robots with limitations	Methods considering limitations and avoiding singularities (Aghili, 2008; Xu et al., 2008)	Achieves smooth and efficient movements while respecting robot limitations	May require more complex planning algorithms compared to simpler methods
Limited base movement for under-actuated robots	A new method for simpler calculations with smooth point-to-point movements (Agrawal et al., 2009)	Reduces computational complexity for under-actuated robots	May limit maneuverability for complex tasks
Complex environments	CHOMP method refining paths for smoothness (Ratliff et al., 2009)	Improves smoothness of existing paths for various robots	Can be computationally expensive for highly cluttered environments
Force limitations while stopping a spinning satellite	Two-step approach with the fastest slowing method and control system (Aghili, 2009)	Optimizes spin-stopping process for efficiency and safety	Requires accurate modeling of satellite properties and limitations
Re-orienting the base after docking	Using only arm movements for docking and re-orientation (Xu et al., 2009)	Reduces reliance on thruster fuel	May require more complex arm movements and planning
Uncertainties during object capture with vision	Predicting object movement for smooth capture path (Aghili, 2012)	Improves capture success rate in uncertain environments	Requires robust vision systems and processing power for real-time prediction
Keeping the robot stable during the capture of the spinning target	Minimizing capture disturbances for smoother and safer capture (Flores-Abad et al., 2014)	Reduces risks of instability during capture	May require additional sensors or advanced control algorithms
Stabilizing robot with tethered object	Combining arm movements, tethers, and jets for smooth stabilization (Wang et al., 2015)	Enables manipulation of objects with tethers	Requires complex control systems and coordination between different actuators
Unknown properties of the captured object	Control method adjusting arm motions for unknown objects (Nguyen-Huynh and Sharf, 2013)	Functions even without detailed object data	May require additional sensors or assumptions about object properties
Momentum of large, captured objects	Two-stage control method for reducing momentum and distributing remaining for stability (Zhang et al., 2017)	Mitigates risks from object momentum	May require more complex control algorithms and precise execution
Limited information about spinning objects	Two-step optimization process for controlling movement and rotation (Virgili-Llop et al., 2017)	Improves planning for objects with unknown spin characteristics	Requires advanced optimization techniques and computational resources
The energy use for robots with arms	Planning method breaks down planning into smaller problems (Misra and Bai, 2017)	Optimizes energy consumption for robots with arms	

### 5.1 Challenges of motion planning in space

Motion planning, the process of guiding a robot through its environment, is particularly challenging in space due to several unique factors. These challenges must be addressed to ensure the success of OOS missions, where precise and reliable motion planning is paramount.

• Microgravity: Unlike Earth, where gravity provides a constant reference for planning, robots in space experience near-weightlessness. This necessitates alternative methods for determining orientation and accounting for the absence of frictional forces (Papadopoulos, 1992). The lack of gravity significantly impacts how robots move and orient themselves, requiring planners to consider these factors when designing trajectories.
• Unmodeled Environments: Space is vast and constantly changing, with asteroids, debris, and unmapped regions posing potential hazards. Planning algorithms must be robust enough to handle unforeseen obstacles and limited environmental data (Kuffner and LaValle, 2000). The unpredictability of the space environment means that motion planners need to be adaptive and resilient, capable of adjusting to new information as it becomes available.
• Computational Constraints: Onboard computers on spacecraft have limited processing power compared to ground-based systems. Motion planning algorithms need to be efficient and require relatively low computational resources to function in real-time (Tortopidis and Papadopoulos, 2007). This constraint makes it essential to develop algorithms that can operate within these limitations while still providing reliable guidance for the spacecraft.

These challenges necessitate specialized planning approaches tailored to the unique conditions of space. The following sections will delve into the strategies developed specifically for spacecraft motion planning, highlighting how these techniques address the challenges outlined above.

### 5.2 Strategies for spacecraft motion planning

Given the challenges of motion planning in space, robust and efficient strategies have been developed to guide spacecraft safely and effectively. These strategies are critical for ensuring the success of OOS missions, where precision, safety, and resource management are top priorities.

• Trajectory Optimization: Fuel efficiency is paramount in space due to the high cost of propellant. Optimization algorithms generate fuel-efficient paths by considering orbital mechanics and thruster limitations (Misra and Bai, 2017). These algorithms break down complex maneuvers into smaller, more manageable steps, optimizing fuel consumption throughout the entire trajectory. Efficient fuel use is crucial for extending mission lifespans and enabling more complex operations.
• Docking Maneuvers: Safely approaching and connecting with another spacecraft requires precise planning. Algorithms factor in relative positions, velocities, and potential thruster firings to create collision-free trajectories for docking (Xu et al., 2009). In some cases, these algorithms may even consider using only the robot’s arms for docking and re-orienting the spacecraft after attachment, minimizing thruster fuel usage. Effective docking maneuvers are vital for the success of OOS missions, where precision and safety are non-negotiable.
• Collision Avoidance: Spacecraft operate in environments with micrometeoroids and orbital debris. Planning algorithms incorporate sensor data and dynamic obstacle avoidance techniques to ensure safe navigation (Kuffner and LaValle, 2000). This can involve rapidly replanning trajectories in real-time to avoid unforeseen obstacles or creating safe zones within the environment that the spacecraft should avoid entirely (Papadopoulos, 1992). Collision avoidance is essential for protecting both the spacecraft and its surroundings during OOS operations.

These strategies form the foundation for the safe and efficient movement of spacecraft. By addressing the specific challenges of motion planning in space, these techniques ensure that OOS missions can be carried out effectively, even in the most demanding conditions.

### 5.3 Manipulation of objects in space

While spacecraft motion planning focuses on the vehicle itself, a crucial aspect of space robotics involves the manipulation of objects. Manipulation in space introduces additional challenges due to the unique properties of the space environment and the objects encountered.

• Uncertain Object Properties: Robots often encounter objects with unknown mass, distribution, or material properties. Planning algorithms need to be flexible and adapt to unforeseen characteristics of the object during manipulation (Abiko et al., 2006; Nguyen-Huynh and Sharf, 2013). This flexibility is critical for ensuring successful capture and manipulation, particularly when dealing with non-cooperative or unknown objects.
• Spinning Objects: Grasping and maneuvering a spinning object introduces gyroscopic forces that can destabilize the robot. Planning methods incorporate spin dynamics and control strategies to ensure safe capture and stabilization (Aghili, 2009; Dimitrov and Yoshida, 2004). Techniques such as approaching the object from a specific axis to minimize spin or using reaction wheels or thrusters to counter the gyroscopic effect are employed to handle these challenges. Effective management of spinning objects is crucial for the success of OOS missions involving debris capture or satellite repair.
• Tethered Objects: Some tasks involve objects connected by tethers to the spacecraft or robot. Planning algorithms need to consider the tether’s dynamics and avoid entanglement during manipulation (Wang et al., 2015). Coordinating arm movements with tether deployment and retraction or using control systems that account for the tether’s influence on the overall system ensures safe and effective operations. Tether management is particularly important for OOS missions where the safe retrieval or deployment of objects is required.

The ability to manipulate objects in space is a fundamental capability for OOS missions. The strategies developed for handling the unique challenges of space manipulation are essential for the success of these missions, enabling robots to perform complex tasks such as satellite repair, debris removal, and in-orbit assembly.

### 5.4 Optimization for performance

In space missions, efficiency and safety are paramount. Motion planning algorithms must consider various performance metrics to optimize robot maneuvers, balancing competing priorities to achieve mission objectives.

• Fuel Consumption: As mentioned previously, propellant is a precious resource in space. Optimization algorithms can prioritize fuel efficiency by generating trajectories that minimize thruster usage (Misra and Bai, 2017). This may involve planning maneuvers that leverage orbital mechanics or utilizing low-thrust, high-efficiency engines for specific mission phases. Efficient fuel use is critical for extending the operational lifespan of spacecraft and enabling more complex missions.
• Maneuvering Time: In some scenarios, completing a task swiftly is crucial. Time-optimal motion planning algorithms prioritize speed while ensuring safety (Kuffner and LaValle, 2000). This may be important for tasks such as satellite collision avoidance or spacecraft reorientation before critical events. Swift maneuvering is essential for responding to dynamic situations in space, where delays can have significant consequences.
• Smoothness and Stability: Minimizing jerky motions is essential for delicate tasks or to reduce wear and tear on the robot’s actuators. Smoothness-based planning algorithms prioritize generating trajectories with minimal accelerations and decelerations (Ratliff et al., 2009). This can improve the precision of manipulation tasks or ensure a smoother ride for astronauts onboard the spacecraft. Smooth and stable operations are vital for maintaining the integrity of both the spacecraft and its mission objectives.

These performance metrics are often competing priorities. For instance, a fast trajectory movement might require more fuel or introduce excessive jerks. The planning algorithm needs to be configured to balance these objectives based on the specific mission requirements. By optimizing for performance, motion planners ensure that OOS missions can be carried out efficiently and safely, meeting the stringent demands of space operations.

### 5.5 Real-time planning for unforeseen situations

The environments robots operate in can be unpredictable, and space is no exception. This section explores the challenges and approaches for motion planning when encountering unforeseen situations or when new information becomes available during a mission.

**FIGURE 13:**
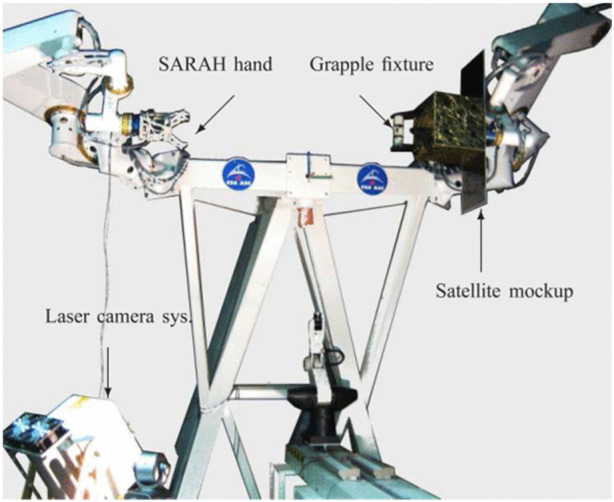
Two manipulator arms mimic satellite motion and autonomously capture the mockup satellite using SARAH (Laliberte and Gosselin, 2001) robotic hand (Aghili, 2012).

• Limited Onboard Processing: Spacecraft computers have limited computational resources compared to ground-based systems. Planning algorithms need to be efficient enough to replan trajectories in real-time without excessive delays (Tortopidis and Papadopoulos, 2007). His may involve breaking down complex planning problems into smaller, more manageable chunks that can be solved quickly onboard. Efficient real-time processing is crucial for adapting to dynamic conditions in space, where rapid decision-making is often required.
• Trade-off Between Optimality and Flexibility: While pre-mission planning can optimize trajectories for anticipated scenarios, real-time situations may demand flexibility. Planning algorithms need to strike a balance between finding optimal solutions and adapting to new information (Aghili, 2012). As illustrated in Figure 13, the experimental setup with two manipulator arms, one equipped with the SARAH robotic hand, demonstrates the ability to autonomously capture a satellite mockup, highlighting the need for adaptability in response to dynamic conditions during on-orbit operations. Flexibility in planning is essential for responding to unforeseen challenges, ensuring that the mission can continue even when conditions change.
• Sensor Integration and Feedback Control: Real-time planning relies heavily on sensor data to perceive the environment and adapt the plan. Algorithms need to integrate sensor data from cameras, lidar, and other sensors to update the environment model and replan the trajectory (Flores-Abad et al., 2014). Feedback control systems can further refine the robot’s motion based on real-time sensor readings, ensuring continued safety and task achievement. Effective sensor integration and feedback control are vital for maintaining the accuracy and reliability of OOS operations, particularly in dynamic environments.

The development of robust real-time planning algorithms is critical for autonomous space robotics. By ensuring that robots can adapt to unforeseen situations, these algorithms enhance the reliability and success of OOS missions, allowing for continued operations even in the most challenging conditions.

## 6 Feedback control

**FIGURE 14:**
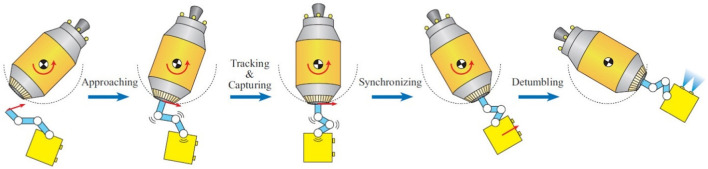
Phases of capturing a tumbling targe (Hirano et al., 2017).

This section explores various control methods used for robots with manipulators (arms) operating in space. These methods address the unique challenges of the space environment, such as microgravity, object manipulation, and uncertainties. As depicted in Figure 14, capturing a rotating target using a robotic arm involves multiple steps, including approaching, tracking, capturing, synchronizing, and detumbling, each of which must be carefully controlled to manage the complexities of space operations. The effectiveness of these control methods is critical for the success of On-Orbit Servicing (OOS) missions, where precision and reliability are paramount.

A summary of control tools and methods in this section can be found in Table 5.

**TABLE 5:** A Summary of methods and tools in feedback control.

Method/Tool	Description	Pros	Cons
Planning Movements for Free-Flying Robots (Papadopoulos and Dubowsky, 1991)	Considers both arm movement and spacecraft stability for robots with free-flying bases	-Enables coordinated motion for complex tasks	-Requires complex modeling and computational power
Multiple Impedance Control (MIC) (Moosavian and Papadopoulos, 2010)	Improves cooperation between multiple robot arms for object handling	- Ensures smooth and stable interactions during manipulation. - Adapts to object weight and forces	- Requires accurate object property estimation
Simulation of Robot-Object Interaction (Ma, 1995)	Develops simulations for complex interactions between robots and objects	- Analyzes pre-mission for potential challenges - Considers complex shapes, contacts, and materials	- May not perfectly replicate real-world interactions
Impedance Control (Yoshida et al., 2004)	Adjusts robot stiffness for object handling tasks	- Enables smoother grasping and manipulation - Reduces risk of object damage	- Requires careful tuning of impedance parameters
Neural Networks for Robust Control (de et al., 2006)	Integrates neural networks with traditional control for handling uncertainties	- Improves robustness to unexpected conditions	- Can be computationally expensive - Requires training data
Model-Based Predictive Control with Reaction Dynamics (Rekleitis and Papadopoulos, 2014)	Analyzes a robot’s control system beforehand for robustness to unexpected object properties	- Predicts performance under changing conditions	- Requires accurate robot and object models
Sliding Mode Control (Ghosh Dastidar, 2010)	Maintains position and attitude control during spacecraft maneuvers	- Simple and robust to uncertainties	- May require high control effort and fuel usage
Friction Compensation (CHEN et al., 2011)	Reduces friction effects in complex joints and gears for precise control	- Improves position and force control accuracy	- Requires specialized hardware and may increase complexity
Multiple Wrist Control for Soft Capture (Uyama et al., 2012)	Uses a special wrist on the robot arm for gentle object capture	- Minimizes impact forces during grasping	- Requires additional hardware complexity
Kalman Filter and Model Reference Adaptive Control (Espinoza and Roascio, 2017)	Estimates unknown robot properties and adjusts control in real-time	- Adapts to unexpected situations	- May require significant computational resources
Dynamic Modularity (Wang et al., 2017)	Adapts existing control systems for free-flying robot arms with limited modification possibilities	- Works with existing control systems	- May not achieve optimal performance compared to redesigned systems
Hardware-in-the-Loop (HIL) Testing (Mou et al., 2018)	Allows testing and refining robot designs before spaceflight in a ground-based environment	- Reduces risks associated with space missions - Improves overall robot intelligence and autonomy	- May not perfectly replicate all space conditions
Spring-like Control for Safe Contact (Flores-Abad et al., 2018)	Absorbs impact forces during robot-object contact	- Reduces risk of damage to robots and captured objects	- May introduce additional complexity to the control system
Online Path Planning and Compliance Control (Hirano et al., 2018a)	Combines real-time path adjustments with compliant control for capturing moving objects	- Enables grasping of moving targets - Reduces contact forces	- Requires significant processing power for real-time path adjustments
Learning Algorithm for Object Identification (Zong et al., 2019)	Analyzes robot movements to identify properties of a captured object	- Reduces need for prior object information	- May require time to converge on accurate identification

### 6.1 Motion planning and coordination for spacecraft-robot systems

Coordinating robot arm movements with the stability of the spacecraft is crucial for successful operations in space. Traditional motion planning approaches often overlook the potential disruption that a moving arm can cause to the spacecraft’s center of mass. To address this challenge, researchers have developed planning algorithms that consider both obstacle avoidance and spacecraft stability during robot arm maneuvers (Papadopoulos and Dubowsky, 1991). By separating the control of the spacecraft’s center of mass, rotation, and arm position, these strategies allow for more efficient fuel usage and smoother, more coordinated movements for both the robot arm and the spacecraft (Giordano, 2020; Giordano et al., 2020).

Recent advancements have further enhanced these approaches by integrating model-based (MB) controllers with model predictive controllers (MPC) to improve the robustness and accuracy of motion planning for free-floating space manipulator systems (SMS). This hybrid MB/MPC method is computationally efficient and effective in handling parametric uncertainties, disturbances, and sensor noise, as demonstrated in numerical simulations (Psomiadis and Papadopoulos, 2022). The integration of these advanced control strategies ensures that OOS missions can be conducted with greater precision and reliability, even in the face of complex environmental challenges.

Furthermore, the growing interest in autonomous multi-robot systems for space applications has led to the exploration of Nonlinear Model Predictive Control (NMPC) for kinematically redundant space robots. Unlike traditional high-level planning methods, NMPC can incorporate collision avoidance directly into the control constraints, providing real-time optimization of the robot’s trajectory within a constrained workspace. Simulation studies have demonstrated the effectiveness of NMPC in maintaining control over a 7-DOF manipulator mounted on a 6-DOF free-floating spacecraft, highlighting its potential for complex space operations (Kalaycioglu and De Ruiter, 2023a; Wang et al., 2016). These developments are paving the way for more sophisticated and autonomous OOS missions, where robots can perform tasks with minimal human intervention.

### 6.2 Cooperative manipulation in space robotics

Achieving smooth and stable manipulation with multiple robot arms in space presents a unique challenge. Unlike terrestrial environments, where robots can rely on a stable base and predictable forces, space introduces additional complexities such as microgravity and the need for precise coordination to prevent collisions and maintain object stability during tasks. To address these challenges, researchers have developed control methods that focus on the object being manipulated rather than the individual arm movements (Schneider and Cannon, 1992).

One significant advancement in this area is the development of multiple impedance control (MIC) techniques, which consider the object’s weight and external forces to ensure smoother interactions between the robots and the manipulated object (Moosavian and Papadopoulos, 2010). These advancements, along with recent work on Optimal Control Allocation (OCA) and Nonlinear Model Predictive Control (NMPC) for dual-arm coordination, enable more sophisticated cooperative manipulation tasks in space environments (Kalaycioglu and De Ruiter, 2023b).

Building on these advancements, new research has demonstrated the effectiveness of NMPC strategies in real-time trajectory tracking and collision avoidance for kinematically redundant space robots. By translating collision avoidance into control constraints, these strategies improve the system’s ability to operate in complex environments with multiple obstacles, making them highly suitable for OOS missions where precision and safety are critical.

### 6.3 Overcoming challenges in object simulation and interaction for space robots

Simulating and interacting with objects in space poses unique challenges for robot control systems. Unlike controlled environments on Earth, where objects and forces can be more easily predicted and managed, space introduces complexities such as irregular object shapes, multiple contact points, and the potential for flexible materials. These factors can significantly impact the accuracy and effectiveness of robot control systems during OOS missions.

To address these challenges, researchers have developed new simulation methods capable of accurately modeling interactions in the space environment. These advancements enable more precise robot control during object manipulation tasks, ensuring that OOS robots can perform their duties effectively even when dealing with complex and unpredictable objects (Ma, 1995). The ability to accurately simulate and manage these interactions is crucial for the success of OOS missions, where precise control is required to safely and efficiently manipulate objects in space.

### 6.4 Capturing moving objects with space robots

Capturing objects in motion is one of the most critical tasks for space robots, requiring precise control to avoid jerky movements and ensure a smooth, stable grasp. This task is particularly challenging in the space environment, where microgravity and the lack of external reference points make it difficult to predict and control the movement of both the robot and the target object.

Two key challenges in this area are coordinating movements for a smooth grasp and matching gripper stiffness to the object’s properties. Robots need to anticipate the object’s trajectory and adjust their movements accordingly to avoid sudden stops or jerks during capture (Ali et al., 1997).

Additionally, the gripper’s stiffness must be carefully controlled to accommodate the object’s properties; a gripper that is too stiff can cause the object to bounce away, while one that is too loose might not secure the object properly (Yoshida et al., 2004).

**FIGURE 15:**
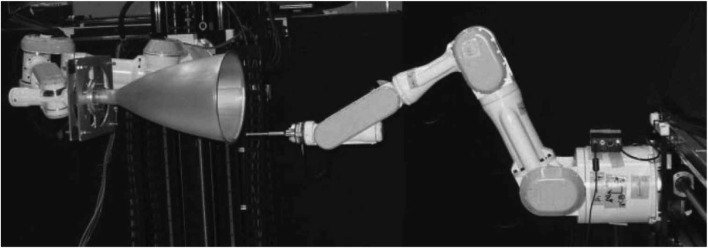
Satellite capture experiment with a nozzle cone (Yoshida et al., 2004).

Researchers have addressed these challenges by developing control methods based on different mathematical models for spacecraft with multiple robot arms (Ali et al., 1997). Additionally, impedance control with “virtual mass” has been proposed to dynamically adjust gripper stiffness, ensuring a secure and smooth grasp of the moving object (Yoshida et al., 2004). These advancements are critical for enabling space robots to effectively capture and manipulate moving objects during OOS missions, as demonstrated Figure 15, where a satellite capture experiment using a nozzle cone is depicted.

Further advancements in this area include the development of a control system that integrates trajectory planning with model predictive control (MPC) for satellite-mounted manipulators. This system improves the precision of capture maneuvers, particularly in scenarios involving parametric uncertainties and disturbances (Rybus et al., 2018). Additionally, the Modified Impedance Control (MIC) concept, originally developed for real-time collision avoidance and manipulation in unstructured environments, offers a powerful approach for dynamically adjusting robot trajectories. This concept allows the robot to maintain collision-free trajectories while incorporating the robot’s dynamics, constraints, and the avoidance of singular configurations, making it highly suitable for real-time applications in space (Kalaycioglu et al., 1993).

### 6.5 Sensor calibration and uncertainty handling in space robotics

The unforgiving environment of space presents unique challenges for robot sensor calibration and uncertainty handling. Unlike their Earth-bound counterparts, robots in space often lack the luxury of readily available calibration tools or controlled environments, making it difficult to maintain the accuracy and reliability of sensor data.

One major challenge is calibrating sensors without additional tools. Traditional methods rely on specialized equipment that is simply unavailable in the vast expanse of space (Aghili, 2000). To overcome this, researchers have developed innovative approaches that allow robots to self-calibrate using their own movements, effectively making them self-sufficient in maintaining sensor accuracy.

Another significant hurdle is dealing with unexpected forces during operation. Unforeseen disturbances during robot arm movements can disrupt sensor readings, leading to control errors (Aghili et al., 2001). Control system adjustments in real-time can improve stability and minimize unwanted interactions caused by these unexpected forces.

Finally, uncertainties about the robot or object’s properties can also cause problems. Lack of precise information about the robot’s characteristics or the target object’s properties can lead to control errors (Abiko and Yoshida, 2010; Christidi-Loumpasefski et al., 2020; Espinoza and Roascio, 2017).

Advanced control techniques based on “reaction dynamics” or system identification with Kalman Filters can compensate for these uncertainties, improving control accuracy despite the challenging conditions (Abiko and Yoshida, 2010; Espinoza and Roascio, 2017). Additionally, new methods allow robots to quickly identify their own properties without prior information, further enhancing control accuracy (Christidi-Loumpasefski et al., 2020).

These advancements allow robots in space to operate effectively despite the inherent uncertainties of their environment, ensuring that OOS missions can be carried out with the necessary precision and reliability.

### 6.6 Advanced control techniques for space robotics

In addition to the conventional control techniques discussed in previous sections, advanced methods such as Optimal Control Allocation (OCA) and Nonlinear Model Predictive Control (NMPC) are being explored to address the unique challenges faced by space robotics. These techniques are particularly useful in scenarios where the system is underdetermined or involves complex dynamics, such as multi-rover systems with dual-arm manipulators.

The OCA technique focuses on minimizing a quadratic cost function related to the torques, contact forces, and moments in the system, ensuring that control inputs are optimized for efficiency and effectiveness. Meanwhile, the NMPC approach accounts for both current and future state estimates to optimize control inputs over a specified prediction horizon. Recent research has shown that while NMPC is computationally more intensive, it provides superior results in minimizing joint and wheel torques, as well as contact moments and forces, especially when dealing with large payloads in space (Kalaycioglu and Ruiter, 2023). Moreover, the introduction of passivity-based NMPC (PNMPC) for multi-robot systems in space offers a novel approach to ensure closed-loop stability while maintaining high performance (Kalaycioglu and De Ruiter, 2023a).

These advanced control techniques are critical for addressing the increasingly complex demands of space robotics, ensuring that OOS missions can be conducted safely and efficiently, even in the most challenging scenarios.

### 6.7 Specific applications of space Robot Arm Control

Space robots play a crucial role in various tasks, and their control systems need to be tailored to the specific application. Here are some prominent examples:

• Space Stations (ISS): The Special Purpose Dexterous Manipulator (SPDM) on the International Space Station (ISS) is designed for delicate tasks requiring high precision control (Mukherji and Rey, 2001).
• On-Orbit Servicing (OOS): Robots used for on-orbit servicing of satellites require specialized features and remote-control capabilities to handle complex maintenance tasks (Landzettel et al., 2006).
• Docking Control: Minimizing unnecessary movements during satellite docking maneuvers ensures a stable connection and efficient fuel usage (Hiramatsu and Fitz-Coy, 2007).
• Building Large Space Structures: When constructing large structures in space, control systems need to separate the movements of fast-moving robots from the slower-reacting structure itself to minimize vibrations (Boning and Dubowsky, 2010).
• Satellite Capture: Capturing a satellite with a robot arm presents a unique challenge. Control methods need to be designed to prevent the robot from pushing the satellite away during contact (Ma et al., 2015; Nakanishi and Yoshida, 2006; Stolfi et al., 2017; Uyama et al., 2012).
• Tumbling Object Capture: Grasping a rapidly spinning object requires advanced control systems that account for sensor errors, delays, and the object’s unpredictable movements (Hirano et al., 2017; 2018a; Wu et al., 2017).
• Robot Arm Control with Uncertainties: Analyzing the control system’s ability to handle unexpected changes in the environment is crucial for safe and reliable robot operation (Rekleitis and Papadopoulos, 2014; Wang et al., 2017).

By tailoring control systems to the specific application, robots can perform a wider range of complex tasks in the demanding environment of space, ensuring that OOS missions can be conducted safely and effectively.

## 7 Application of Machine Learning

The burgeoning field of on-orbit servicing (OOS), encompassing tasks like satellite repair and in-space assembly, promises to extend the life and capabilities of spacecraft. However, current approaches often rely on rigid pre-programming and ground-based control, limiting their ability to handle the complexities and uncertainties of space operations. Machine learning (ML) offers a paradigm shift, empowering spacecraft with the ability to learn, adapt, and make real-time decisions autonomously. This section delves into how ML is revolutionizing OOS, enhancing the precision, efficiency, and adaptability of these missions. By exploring how ML algorithms are applied in various aspects of OOS, we can glimpse a future where intelligent spacecraft perform complex tasks with greater autonomy and robustness.

Executing neural network (NN)-based software on orbit introduces significant energy demands, a challenge that is particularly acute in the constrained environment of space. To mitigate this issue, edge and cloud computing approaches are increasingly being integrated into space missions. Edge computing allows for processing data closer to where it is generated, reducing the need for constant communication with Earth and conserving energy. Meanwhile, cloud computing offers a scalable solution for handling the immense computational loads associated with NN algorithms, offloading some processing tasks to ground-based servers or distributed networks in orbit. By leveraging these technologies, spacecraft can maintain the high performance of ML-driven operations while efficiently managing their energy resources, enabling longer mission durations and enhanced capabilities (Huang et al., 2020).

A summary of methods and tools found in this section can be found in Table 6.

**TABLE 6:** A Summary of methods and tools in Machine Learning.

Method/Tool	Notes
Neural networks for controlling robot arms	Trains the robot arm directly on the robot as it moves, leading to significant improvement in accuracy and adaptability to unexpected situations (Newton and Xu, 1993a; Newton and Xu, 1993b)
Adaptive NN controllers for formation flying	Improves spacecraft control in formation flying by adapting to uncertainties. The first system can be fooled, but the second system avoids this issue and reduces control effort (Zou and Kumar, 2011)
Fuzzy logic control system for spacecraft	Improves spacecraft control by using a new type of fuzzy membership function. This function allows for better separation between the fuzzy logic’s core aspects and its uncertainty handling. The method ensures stability and improves performance (Khanesar et al., 2015)
Neural network combined with control system design for spacecraft	Controls a spacecraft with weak thrusters by learning its properties during flight. This allows the system to reuse the learned information for similar missions later, saving time and effort (Zeng and Wang, 2015)
Learning system for small space robots	Improves the precision of small space robots during delicate close-up tasks by combining real-time adjustments with lessons learned from past movements (Ulrich and Hovell, 2017)
Convolutional Neural Network (CNN) for spacecraft attitude control	Handles unexpected problems like faulty thrusters, outside forces, and unpredictable movements of the spacecraft itself. The neural network learns and adapts to these issues in real-time (Cao et al., 2018)
Soft Q-learning for robot arm training	Trains robots with one or two arms to capture objects in space without needing a perfect model of the robot by rewarding them for success and encouraging random exploration (Yan et al., 2018)
Convolutional Neural Network (CNN) for object pose estimation	Estimates a 3D object’s position and orientation (pose) in space using just a single image. This method is effective for space missions due to its ability to handle constantly changing light conditions (Hirano et al., 2018b)
Two-layer control system for formation flying	Controls multiple spacecraft flying together by first setting performance goals and then adding a learning-based control system that adapts to unexpected issues. This adaptation happens in real-time without needing any prior information about the spacecraft themselves (Stolfi et al., 2019; Wei et al., 2019)
Deep Neural Networks for robot arm control on free-floating spacecraft	Learns how to control the robot based on data, including the flexibility and friction of the joints. This allows the robot to follow a planned path while considering these complexities (Stolfi et al., 2019)
Deep Deterministic Policy Gradient for robot arm control	Controls a free-flying robot arm to capture objects in space without needing a complex model of the robot. It uses a pre-training step to improve learning efficiency (Du et al., 2019)
Reinforcement Learning for spacecraft attitude control	Controls a spacecraft’s orientation by using a machine learning technique called Reinforcement Learning. This method avoids complex calculations and refines the controls over time to further improve performance (Allison et al., 2019; Ramadan and Younes, 2019)
Combining machine learning and 3D modeling for object pose estimation	Determines a satellite’s position and orientation (pose) from a single image. A machine learning program finds specific features on the satellite’s image, then uses a pre-built 3D model to calculate the satellite’s exact pose (Chen et al., 2019)
Two-step convolutional neural network (CNN) for object pose estimation	Finds a spacecraft’s position and direction (pose) from a single image, even if the spacecraft is not cooperating. This program disguises the training images with different textures to improve its generalizability (Park et al., 2019)
Combining control methods, artificial intelligence, and external bump estimation for robot arm control	Controls a free-flying robot arm in space with uncertainties and external bumps by combining three techniques: 1) a special control method to reduce jittering, 2) artificial intelligence to handle uncertainties, and 3) a technique to estimate external bumps (Xie et al., 2020)

### 7.1 Sensing of pose and state

Accurate knowledge of a spacecraft’s pose (position and orientation) and state (dynamics) is critical for various OOS tasks, including docking, manipulation, and inspection. Traditionally, this information is obtained through a combination of sensors (cameras, LiDAR) and complex mathematical models. However, ML offers a powerful alternative, particularly when dealing with complex or unstructured data, such as images from space. The integration of ML into pose and state estimation processes is transforming how spacecraft perceive and interact with their environment, leading to more reliable and efficient OOS operations.

**FIGURE 16:**
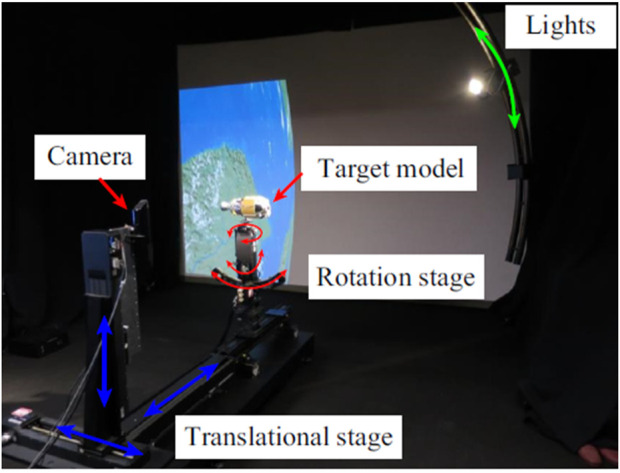
Hardware simulator using realistic pictures to evaluate the trained model (Hirano et al., 2018b).

This subsection explores recent advancements in using ML for pose and state estimation in OOS. A key challenge in space applications is the variability of lighting conditions, which can significantly affect sensor readings. (Hirano et al., 2018b). addressed this issue by employing a Convolutional Neural Network (CNN) to directly estimate an object’s pose from a single image. Their CNN was trained on simulated data encompassing various lighting scenarios (Figure 16), demonstrating the effectiveness of simulation-based training for real-world tasks. This approach exemplifies how ML can overcome the limitations of traditional methods in dynamic and unpredictable environments.

Another approach leverages a combination of ML and existing 3D models. (Chen et al., 2019). presented a method that utilizes ML to identify specific features on a satellite’s image, followed by pose calculation based on a pre-built 3D model. This strategy achieved high accuracy and highlights the potential of integrating ML with established techniques to enhance the reliability of OOS tasks. Such hybrid approaches are becoming increasingly important as the complexity of space missions grows.

Uncooperative spacecraft, which may not emit identification signals, pose additional challenges. (Park et al., 2019). tackled this by developing a CNN that first locates the spacecraft in an image and then identifies key points for pose estimation using a 3D model. Their method employed data augmentation techniques to improve robustness against unseen images, demonstrating its applicability for non-cooperative targets. This method underscores the adaptability of ML in handling diverse and challenging scenarios in space.

Similar to (Park et al., 2019; Proenca and Gao, 2020) focused on pose estimation during close-proximity maneuvers, where challenging lighting and cluttered backgrounds can hinder traditional methods. They proposed URSO, a system that generates realistic simulated space images for training a deep learning model to analyze real photos and estimate poses. This approach highlights the value of simulated data generation for tasks with complex environmental conditions, paving the way for more robust and adaptable OOS operations.

Beyond pose estimation, ML can also improve the control of space objects. (Huang and Sands, 2023). presented a method that utilizes ML to address inaccurate initial assumptions about an object’s properties. Their approach employs mathematical equations to learn the object’s true properties over time, leading to more precise control, especially when initial information is limited. This dynamic learning capability is crucial for enhancing the autonomy and effectiveness of OOS missions.

These studies highlight the significant potential of ML for pose and state estimation in OOS. As ML algorithms continue to develop and computational resources become more accessible on spacecraft, we can expect even more innovative and robust solutions for future space missions, making OOS operations safer and more efficient.

### 7.2 Feedback control

This subsection explores recent advancements in using ML for feedback control in OOS. Feedback control is vital for maintaining the stability and precision of spacecraft and robotic systems, particularly in the unpredictable environment of space. ML techniques are increasingly being applied to enhance the adaptability and robustness of these control systems, enabling more effective and autonomous OOS missions.

One common challenge in space robotics is controlling flexible robotic arms. (Newton R. T. and Xu Y., 1993; Newton R. Todd and Xu Y., 1993). addressed this by employing a neural network that learns directly on the robot during operation, significantly improving accuracy compared to traditional methods. This real-time learning capability is essential for dealing with the dynamic conditions encountered in space, where pre-programmed solutions may fall short.

Formation flying, where multiple spacecraft maintain a specific configuration, requires robust control systems that can adapt to uncertainties. (Zou and Kumar, 2011). proposed two adaptive neural network controllers that outperform traditional methods by adjusting to unforeseen conditions. This adaptability is crucial for ensuring the success of complex OOS missions involving multiple spacecraft.

Another approach utilizes fuzzy logic control systems, known for handling imprecise data. (Khanesar et al., 2015). enhanced a fuzzy logic control system for spacecraft by introducing a new type of membership function. This function separates the core aspects of fuzzy logic from uncertainty handling, leading to improved stability and performance. Such improvements are vital for maintaining reliable control under the uncertain and variable conditions of space.

Uncertainties can also arise from a spacecraft’s unknown properties or weak thrusters. (Zeng and Wang, 2015). proposed a method combining a neural network with a control system design to address this challenge. The neural network observes the spacecraft’s behavior and learns its characteristics, enabling the control system to maintain stability and achieve the desired trajectory. This learned information can be reused for similar future missions, improving efficiency and reducing the need for extensive pre-mission calculations.

**FIGURE 17:**
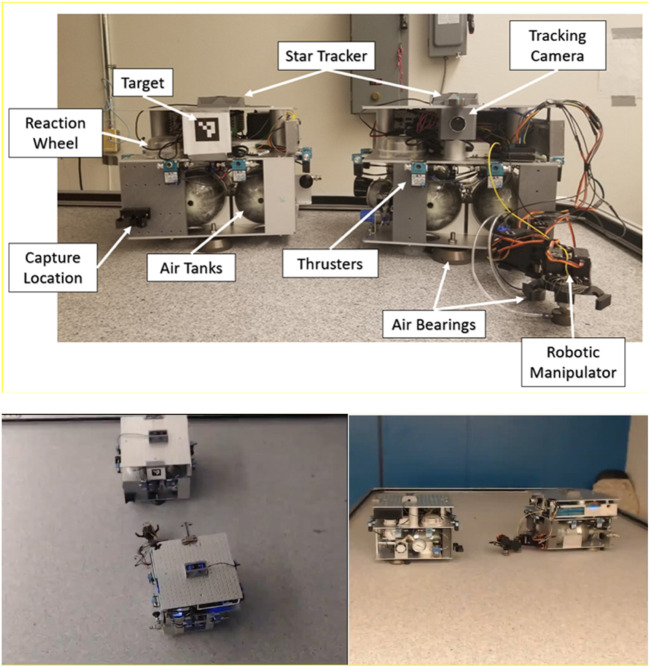
Air-bearing microgravity testbed for autonomous spacecraft rendezvous and robotic capture at York University space Engineering lab (Santaguida and Zhu, 2023).

**FIGURE 18:**
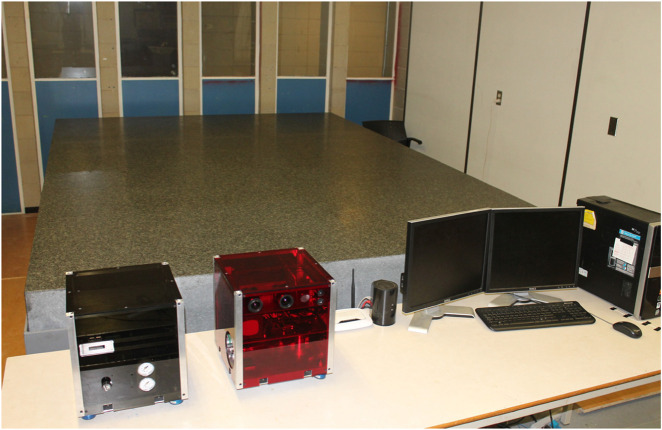
Spacecraft proximity operation testbed at Carleton University. (Ulrich and Hovell, 2017).

Precise control during delicate close-up tasks is particularly critical for small space robots with limited thruster power. Santaguida and Zhu (2023) developed an air-bearing microgravity testbed to validate autonomous control algorithms for spacecraft rendezvous and robotic capture of a free-floating target (Figure 17). (Ulrich and Hovell, 2017) designed a learning system that combines real-time adjustments with past experiences, allowing the robot to progressively improve accuracy without exceeding thruster limitations. Simulations and experiments (Figure 18) confirmed the effectiveness of this method for precise robotic inspection tasks, demonstrating the potential of ML to enhance the capabilities of small, resource-constrained spacecraft.

ML can also enhance spacecraft attitude control, which refers to maintaining a specific orientation. (Cao et al., 2018). introduced a neural network-based approach for attitude control that can adapt to unexpected events like thruster failures, external forces, or even the spacecraft’s own movements. Simulations demonstrated the effectiveness of this method in regulating spacecraft attitude, highlighting its potential for improving the reliability and autonomy of OOS missions.

Training robots for tasks like object capture in space traditionally requires a precise model of the robot’s dynamics. (Yan et al., 2018). proposed a method utilizing soft Q-learning, a type of artificial intelligence, to train robots through trial and error, eliminating the need for a perfect model. Simulations showed that this method allows robots to grasp objects in various configurations, underscoring the flexibility and adaptability that ML can bring to space robotics.

Wei et al. (2019) addressed the challenge of maintaining formation flying amidst uncertainties using a two-layer control system. This system combines predefined performance goals with a learning-based controller that adapts to unexpected situations in real-time, without prior knowledge about the spacecraft. Simulations confirmed this method’s effectiveness in keeping the formation stable, demonstrating the potential of ML to enhance the robustness of complex space operations.

Another complexity arises when controlling robot arms mounted on free-floating spacecraft. These robots can introduce disturbances due to their lightweight and flexible structures. (Stolfi et al., 2019). proposed a method using Deep Neural Networks to learn how to control the robot based on data about its flexibility and joint friction, allowing for precise movement while considering these complexities. This approach is particularly relevant for OOS tasks requiring high precision and stability.

Similar to capturing objects, controlling free-flying robot arms for tasks like grasping also benefits from ML. (Du et al., 2019). introduced a Deep Deterministic Policy Gradient method to train a robot arm for object capture. They incorporated a pre-training step to improve learning efficiency. Simulations confirmed this method’s effectiveness for a 3-degree-of-freedom robot arm, highlighting the advantages of ML in optimizing complex robotic tasks in space.

Unforeseen disturbances and external bumps can hinder the smooth and accurate control of free-flying robot arms. (Xie et al., 2020). addressed this by combining three techniques: a control method for reducing jittering, ML to handle uncertainties, and a technique to estimate external bumps. Simulations validated this method’s ability to achieve smooth and accurate control, demonstrating the value of integrating ML into feedback control systems.

Docking maneuvers can significantly alter a spacecraft’s properties. (Zhao and Duan, 2020). proposed a method that utilizes post-docking data to learn about the new combined system and adjust controls accordingly. This method is particularly useful because it works even with imprecise measurements and unexpected external forces. Simulations confirmed its effectiveness for controlling docked spacecraft, illustrating the adaptability of ML-based control systems in dynamic and complex environments.

Precise spacecraft attitude control is crucial for various missions. (Yao, 2021). proposed a method combining a basic control system with a learning function and a safety net for handling unexpected events. This method offers adaptability while maintaining precise spacecraft orientation, as confirmed through simulations. Such hybrid approaches are becoming increasingly important as space missions grow in complexity.

When a spacecraft captures another object with robotic arms, determining the combined mass and movement properties becomes critical for maintaining control. (Zhao and Duan, 2021). proposed two methods that “learn” these properties and adjust the controls in real time. One method assumes a limit for external forces, while the other even learns about the forces themselves. Both methods work without needing prior knowledge about the captured object and can handle limitations in thruster power. Simulations confirmed that these methods are effective for controlling this newly formed spacecraft combination.

Maintaining spacecraft attitude stability amidst external disturbances is another challenge addressed by ML. (Zhang et al., 2021). proposed an “online-learning control” method that incorporates past control actions into its calculations, allowing for a simpler and more efficient system that achieves good performance in simulations. This approach is particularly valuable for long-duration missions where adaptability and efficiency are crucial.

Zheng et al. (2021) focused on keeping a spacecraft precisely pointed (attitude tracking) during missions with uncertainties. Their method combines a learning component that identifies these uncertainties with a controller that uses both traditional methods and this learning to maintain precise aiming. This method adapts automatically and performs well in simulations, making it promising for various observation missions.

Complex maneuvers, such as attaching to another object, can be challenging to predict mathematically for control purposes. (Liu et al., 2022). proposed a method using reinforcement learning, a type of AI that can learn the best control strategy through trial and error, without a complex mathematical model. This method is promising because it does not require extensive upfront calculations. Simulations confirmed its effectiveness for complex spacecraft maneuvers, demonstrating the potential of ML to simplify and enhance the planning of intricate space missions.

Elkins et al. (2022) explored using reinforcement learning for real-time spacecraft attitude control. This approach allows the spacecraft to “learn” and improve its movements over time, unlike traditional methods with fixed controls. Their research paves the way for more adaptable spacecraft control, potentially leading to more efficient and successful space missions.

Most recently, (Wei et al., 2023), addressed the challenges of maintaining attitude tracking for a rigid spacecraft with uncertainties and thruster limitations. Their method combines two parts:

1. An Inertial-Free Base Controller: This part handles basic attitude control without needing to know the exact properties of the spacecraft.
2.Reinforcement Learning for Tuning: This part fine-tunes the control system for better performance and robustness, even with thruster limitations.

This method offers a simpler implementation than traditional approaches and achieves good performance in simulations, making it promising for real-world spacecraft applications. As evident from these studies, ML offers a powerful toolkit for feedback control in OOS. By adapting to uncertainties and learning from experience, ML-based control systems can enhance the precision, robustness, and efficiency of various space operations.

### 7.3 Spacecraft guidance and navigation

This subsection explores recent advancements in using ML for spacecraft guidance and navigation. ML has the potential to revolutionize these areas by enabling spacecraft to “learn” control strategies through trial and error, leading to more autonomous and resilient systems. As missions become more complex, the ability to adapt and make real-time decisions becomes increasingly critical for the success of OOS.

A key advantage of ML is its ability to learn from experience, which is particularly useful in guidance and navigation tasks. (Hovell and Ulrich, 2020). investigated a deep reinforcement learning method for spacecraft guidance. Unlike traditional methods that require pre-defined strategies, this approach allows the spacecraft to learn the optimal guidance strategy in a simulated environment. Their findings with a simulated docking scenario show promise for developing more autonomous spacecraft guidance systems in the future, where adaptability and real-time decision-making are key.

Uncertainties in spacecraft location data can pose challenges for navigation, particularly during complex maneuvers. (Boone et al., 2022). addressed this issue by training a reinforcement learning program to guide a spacecraft between Earth and the Moon. Their approach incorporates these uncertainties into the training process, equipping the program with information about the range of possible location errors. This allows the program to adapt to unexpected situations during the transfer, making it a more robust solution for spacecraft navigation.

Tipaldi et al. (2022) explored the broader potential of reinforcement learning for various spacecraft control tasks, including landing on other celestial bodies, managing formations of multiple spacecraft, and planning efficient trajectories. Reinforcement learning allows the spacecraft to learn the best control strategies through trial and error, making it adaptable to unforeseen circumstances. Their work highlights the importance of designing effective training simulations and establishing safety and reliability verification methods for AI-based control in spacecraft. Overall, their research suggests that reinforcement learning has the potential to equip future spacecraft with more onboard decision-making capabilities, enhancing the autonomy and resilience of space missions.

This subsection highlights the growing promise of ML for spacecraft guidance and navigation. As these ML algorithms mature and computational resources become more readily available onboard spacecraft, we can expect even more innovative and adaptable control systems for future space exploration endeavors.

### 7.4 Robot motion planning and control

This subsection explores recent advancements in using ML for robot motion planning and control in space. Motion planning is critical for robots operating in the challenging environment of space, where they must perform complex tasks like debris capture, inspection, and assembly. ML provides a way to enhance these capabilities, allowing robots to plan and execute motions with greater precision and adaptability.

**FIGURE 19:**
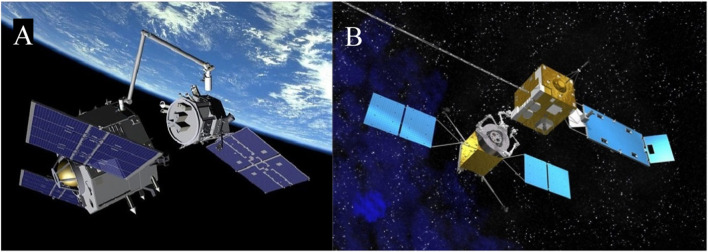
Schematic diagram of **(A)** single arm robot, **(B)** double arm robot (Wu et al., 2020).

A significant challenge involves planning motions for robots with multiple arms operating on a free-flying spacecraft. (Wu et al., 2020). addressed this by employing reinforcement learning to train a two-armed robot to plan its movements in a simulated environment. This approach allows the robot to react to moving targets, unlike traditional offline planning methods that would not work in this scenario. The robot learns through trial and error without requiring a complex model, making it adaptable to unforeseen situations. Figure 19 illustrates the schematic diagrams of single-arm and dual-arm space robots.

Smooth motion planning is crucial for robots with multiple arms, especially in free-floating environments. (Li Y. et al., 2021). tackled this challenge using deep reinforcement learning. Their method allows the robot to learn optimal paths while considering joint limitations and avoiding collisions between the arms. This method proved effective in simulations for both stationary and spinning robots, showcasing the potential of ML to enhance the safety and efficiency of robotic operations in space.

Another challenge involves controlling multiple robot arms for tasks like debris capture. (Jiang et al., 2021). proposed a multi-agent reinforcement learning approach where the arms learn to collaborate and avoid collisions with each other and the target debris during the capture process. Simulations showed this method to be more accurate than traditional methods, making it promising for real-world capture missions and highlighting the collaborative potential of ML in multi-robot systems.

Robots can also learn from human demonstrations, but directly imitating human motions can be inefficient for robots due to their different physical capabilities. (Li C. et al., 2021). proposed a method that combines learning from demonstrations with robot-specific limitations. This method also optimizes the movement plan to reduce jitters and energy use. The authors validated this approach on a ground-based robot imitating a bolt-screwing task, demonstrating the value of combining human expertise with ML to improve robotic performance.

Planning smooth paths for a spacecraft with a robotic arm requires considering various factors like arm movement, torque limitations, and collision avoidance. (Santos et al., 2022). proposed a method that incorporates these factors and utilizes ML to improve planning accuracy and speed. Simulations confirmed that this method is versatile and effective for various scenarios, illustrating the broad applicability of ML in enhancing robotic motion planning in space.

Finally, (Wang et al., 2023), addressed the challenge of controlling a robot arm during object capture in space. The sudden impact when the robot grabs the object can disrupt control. They proposed a method that combines a special control technique to handle the impact with a neural network that adjusts to the captured object’s properties. This method ensures the robot can track the desired motion while adapting to the captured object, underscoring the importance of ML in maintaining precision and stability during dynamic operations.

These studies showcase the significant potential of ML for robot motion planning and control in OOS. As ML algorithms continue to develop and computational resources become more accessible on spacecraft, we can expect even more innovative and robust solutions for robots performing complex tasks in space, ultimately enhancing the autonomy and effectiveness of OOS missions.

### 7.5 Spacecraft learning and autonomy

The ability of spacecraft to learn and operate autonomously is a critical frontier in space exploration. Traditionally, spacecraft rely on pre-programmed commands and predefined control systems. However, ML offers a promising approach for enabling onboard decision-making and adaptation to unforeseen circumstances. As spacecraft become more autonomous, they can perform more complex missions with greater efficiency and resilience, reducing the need for constant human intervention.

This subsection explores recent advancements in using ML to enhance spacecraft autonomy. A key challenge involves controlling a spacecraft’s orientation (attitude) in real time. (Ramadan and Younes, 2019). proposed a method using reinforcement learning, a type of ML that allows the spacecraft to learn through trial and error. This method utilizes simpler models as a foundation for initial control, followed by refinement through ML to optimize performance. This approach holds promise for real-time control of complex systems, as demonstrated in their spacecraft attitude control simulation, highlighting the potential of ML to enhance the autonomy and adaptability of space missions.

Another approach to spacecraft attitude control with ML is explored by (Allison et al., 2019). They investigated using reinforcement learning to train an agent that can consider various scenarios with limitations on the spacecraft’s thrusters or reaction wheels. This method offers adaptability to various spacecraft sizes without reprogramming, unlike traditional methods that require specific spacecraft information. Their findings revealed that reinforcement learning performs slightly better for spacecraft with uncertain properties and achieves similar performance for well-defined spacecraft compared to traditional methods. This suggests that reinforcement learning is a promising new approach for controlling complex spacecraft systems, particularly in unpredictable environments.

Deep space missions pose unique challenges due to unpredictable conditions. (Elkins et al., 2020). addressed this by proposing a deep reinforcement learning method for spacecraft attitude control. This method allows the spacecraft to “learn” how to adjust its movements in real time in response to unexpected forces and disturbances, even if they were not encountered during training. A significant benefit is that this controller can handle various spacecraft designs without needing individual reprogramming. Simulations confirmed the method’s effectiveness for large maneuvers and achieving high pointing accuracy. This research paves the way for more adaptable and robust spacecraft control systems in deep space exploration.

Elkins et al. (2020) also explored developing a reinforcement learning method suitable for less powerful computers, commonly found on many spacecraft. Their system utilizes trial and error to discover the most efficient ways to move the spacecraft, even for large maneuvers. This method achieves pointing accuracy exceeding typical standards. The study demonstrates this concept in a simulated environment and proposes directions for future research to make this system even more practical for real-world spacecraft.

Harris et al. (2022) explored the concept of self-learning spacecraft using deep reinforcement learning for complex missions. Traditionally, spacecraft rely on pre-programmed commands. This new approach allows the spacecraft to learn and adapt its decisions in real time, potentially reducing mission costs. The authors examined various challenges, including translating mission goals into a language the AI understands, managing the complexity of vast amounts of data, and ensuring the safety of these systems. Testing this concept in simulations for tasks like maintaining position and controlling orientation, their findings suggest this method is promising and comparable to other solutions. This research lays the groundwork for AI-powered decision-making becoming a reality for future spacecraft.

In conclusion, these studies highlight the significant potential of ML for enhancing spacecraft learning and autonomy. As ML algorithms mature and computational power on spacecraft increases, we can expect even more innovative approaches for intelligent and adaptable spacecraft operations in the future, leading to more successful and cost-effective space missions.

## 8 Conclusion

This survey has provided a comprehensive overview of the current state-of-the-art techniques in object state estimation, motion planning, and feedback control for On-Orbit Servicing (OOS) robots. By systematically reviewing these areas, we have highlighted the strengths and limitations of existing approaches, particularly in the context of the unique challenges posed by the space environment.

One of the critical findings of this survey is the persistent challenge of ensuring precise and safe manipulation in microgravity, where traditional methods often struggle with uncertainties and dynamic conditions. The integration of Machine Learning (ML) into space robotics, while promising, remains underexplored, with significant computational and reliability challenges that must be addressed before these techniques can be fully leveraged in operational environments.

This survey has identified several key areas where further research is needed. These include improving the robustness of object state estimation under varying space conditions, enhancing motion planning algorithms to better handle real-time adjustments, and advancing the integration of ML techniques into feedback control systems. Addressing these gaps will be crucial for the development of more autonomous and reliable robotic systems capable of performing complex tasks in space.

In conclusion, this survey not only maps out the current landscape of OOS robotic technologies but also provides critical insights into the limitations and areas for future exploration. By identifying these gaps and suggesting directions for further research, we hope to contribute to the ongoing development of space robotics, ultimately enabling more sophisticated and reliable robotic operations in the demanding environment of space.
